# Immunopathogenesis and pathological features of NADC34-like PRRSV infection in pregnant sows during late gestation

**DOI:** 10.1186/s13567-026-01792-0

**Published:** 2026-07-24

**Authors:** Seung-Chai Kim, Hwan-Ju Kim, Sang Chul Kang, Ji-Hyun Ryu, Byungkwan Oh, Sang-Ik Oh, Bumseok Kim, Won-Il Kim

**Affiliations:** 1 College of Veterinary Medicine, Jeonbuk National University, Iksan, Korea; 2 Optipharm Co. Ltd., Cheongju, Korea; 3 Present Address: Department of Microbiology, Icahn School of Medicine at Mount Sinai, New York, NY 10029 USA

**Keywords:** Porcine reproductive and respiratory syndrome virus, immunopathogenesis, abortion, kynurenine pathway, maternal–fetal interface

## Abstract

The recently emerged NADC34-like PRRSV-2 strain (Lineage 1A) has been increasingly linked to severe reproductive failures in sow herds, yet its immunopathogenic mechanisms remain poorly understood, especially in pregnant sow models. In this study, we compared the pathogenic and immunological characteristics of a NADC34-like isolate (JBNU-22-N01) with a prevalent NADC30-like strain (PJ73, Lineage 1C) in pregnant sows during late gestation. JBNU-22-N01 infection resulted in earlier peak viremia, more severe maternal pathology, and greater fetal growth restriction compared with PJ73, despite comparable overall fetal viral loads. Notably, JBNU-22-N01-infected fetuses were overrepresented in high viral burden clusters, suggesting enhanced transplacental viral transmission. Transcriptomic and cytokine profiling at the maternal–fetal interface revealed strain-specific downregulation of epithelial junction genes (e.g., *CLDN1*, *CDH1*) and immune effector genes in JBNU-22-N01 infection, accompanied by elevated local IFN-α but attenuated immune activation. In contrast, PJ73 elicited more robust antiviral transcriptional responses. Additionally, JBNU-22-N01 infection induced a higher serum kynurenine/tryptophan ratio, indicative of systemic activation of the immunosuppressive kynurenine pathway, which may have contributed to local immune dampening at the maternal–fetal interface. Cytokine analyses across maternal and fetal compartments demonstrated compartmentalized regulation, with maternal cytokine expression shaped by viral strain and fetal cytokine responses more strongly correlated with absolute viral burden. Together, these findings suggest that NADC34-like PRRSV impairs maternal barrier and immune defenses via local and systemic modulation, facilitating fetal infection and compromise. This study expands our understanding of immunopathogenic mechanisms driving PRRSV-induced reproductive failure during late gestation and provides insights for future vaccine development strategies.

**Supplementary Information:**

The online version contains supplementary material available at 10.1186/s13567-026-01792-0.

## Introduction

Porcine reproductive and respiratory syndrome virus (PRRSV) is one of the most economically devastating pathogens in the global swine industry, causing an estimated $664 million in annual losses in the USA alone [1]. PRRSV infection leads to reproductive failure in pregnant sows and severe respiratory disease in nursery and growing pigs, with age-dependent differences in clinical outcomes [2]. PRRSV is an enveloped, single-stranded, positive-sense RNA virus belonging to the order *Nidovirales*, family *Arteriviridae*, with a genome size of approximately 15 kb. Two major species of PRRSV are recognized: PRRSV-1 (European lineage, prototype strain Lelystad virus) and PRRSV-2 (North American lineage, prototype strain VR2332), recently reclassified as *Betaarterivirus europensis* and *Betaarterivirus americense*, respectively (ICTV2023).

PRRSV primarily infects porcine alveolar macrophages (PAMs), its primary target cells in the lungs, while showing limited replication efficiency in circulating blood monocytes and monocyte-derived cells [3, 4]. In weaned piglets, PRRSV infection leads to respiratory distress accompanied by interstitial pneumonia, with viremia peaking at 7–12 days post-infection (dpi) and persisting for 4–5 weeks [5]. The virus induces dysregulated immune responses, including impaired T-cell activation and delayed or suppressed adaptive immunity, endangering the host susceptible to secondary infections due to its immunosuppressive effects [5, 6]. In contrast, in older pigs, including gilts and sows, PRRSV infection typically results in lower viremia in both magnitude and duration (up to 2–3 weeks) [7], potentially owing to enhanced innate immune resistance [8]. Rather than respiratory distress, the primary clinical manifestation in pregnant sows is reproductive failure [2, 5], which has significant economic implications for the swine industry.

During early gestation, PRRSV infection may result in embryonic loss or death, depending on the developmental stage of the embryo [9]. While porcine fetuses are susceptible to PRRSV at any stage of gestation when the virus is introduced directly via intra-fetal or intra-amniotic routes, natural transplacental transmission is rare during mid-gestation but becomes more frequent in late gestation [10]. At this stage, PRRSV infection is strongly associated with adverse reproductive outcomes, including abortion, premature farrowing, fetal mortality, and the birth of weak piglets with congenital infection, ultimately increasing preweaning mortality rates [10–12].

However, the precise mechanisms underlying maternal-to-fetal PRRSV transmission remain poorly understood [9]. It is known that PRRSV replication occurs in fetal implantation sites prior to fetal infection, inducing apoptosis in both infected and neighboring cells [13]. Additionally, the abundance of sialoadhesin-positive (Sn^+^, CD169^+^)/CD163^+^ macrophages in the endometrium and placenta, which are permissive to PRRSV, has been implicated as one of the key factors in transplacental viral passage [14]. Once the virus reaches the fetus, PRRSV can be detected in multiple tissues, including the lungs, liver, spleen, heart, and kidneys, with the most consistent viral presence observed in lymphatic tissues. Among these, the fetal thymus is considered a primary site of viral replication [11]. Notably, despite the widespread distribution of PRRSV in fetal tissues, severe microscopic lesions are often absent, suggesting that fetal demise may be driven not by direct viral cytotoxicity [11, 15], but rather by immunopathological disruptions at the maternal–fetal interface (MFI) [2, 16, 17].

In late gestation, when the porcine placenta becomes permeable to PRRSV [2], the fetus enters a phase of rapid growth, requiring substantial structural adaptations in the placenta, including the development of placental folds to increase the MFI area and ensure efficient nutrient transport [18–20]. Tight junctions (TJs) are critical intercellular structures in epithelial and endothelial tissues, forming selective paracellular barriers that regulate molecular transport and maintain compartmentalization [21, 22]. Each TJ-associated protein plays a distinct role: claudins (CLDNs) contribute to TJ formation and paracellular barrier function, occludin (OCLN) regulates junctional permeability, and junctional adhesion molecules (JAMs) interact with scaffolding proteins such as tight junction protein 1 (TJP1; also known as zonula occludens-1, ZO-1) [21, 22]. PRRSV infection has been reported to disrupt TJ integrity in both the endometrium and placenta, with progressive alterations correlating with increasing viral load and fetal preservation status [20, 23], ultimately affecting fetal viability.

Transcriptomic studies have provided crucial insights into PRRSV-induced tissue-specific immune responses and gene expression changes at the MFI and within fetal tissues [16, 17]. While the endometrial immune response was largely adaptive, characterized by the upregulation of humoral and cell-mediated immunity genes, the fetal thymic response was predominantly innate, driven by the strong activation of type I interferon signaling and proinflammatory cytokine pathways [17, 24]. Notably, gene expression in the thymus has been shown to more accurately reflect fetal viral load, whereas placental gene expression serves as a stronger predictor of fetal demise, highlighting the distinct immunological roles of these tissues during PRRSV infection [16]. The placental response played a pivotal role in determining fetal outcome, as dysregulation of apoptosis and ubiquitination pathways has been observed in the placenta of PRRSV-infected fetuses, particularly in those exhibiting meconium staining, a hallmark of fetal compromise [2, 16, 24]. Meconium-stained fetuses displayed a significant upregulation of cytokine and granulocyte-associated genes, indicative of a heightened inflammatory state linked to disease progression [17]. These findings suggest that pathology and immune dysregulation within MFI are key contributors to PRRSV-induced fetal compromise, underscoring the need for further investigation.

In summary, reproductive PRRS models over the past decade have highlighted that vertical transmission is strongly gestation-stage dependent and that early events at the MFI precede overt fetal infection. Collectively, these studies indicate that MFI inflammation and tissue-specific immune dysregulation, rather than direct fetal cytopathology, are major determinants of fetal compromise and provide a framework for interpreting strain-dependent differences in reproductive outcomes.

PRRSV-2 exhibits significant genetic diversity, leading to its classification into 11 genetic lineages (L1–L11) and 21 sublineages (L1A–L1F, L1H–L1J, L5A–L5B, L8A–L8E, and L9A–L9E) on the basis of ORF5 phylogenetic analysis [25]. Among these, NADC34-like PRRSV (Lineage 1A, L1A) has recently emerged as a highly virulent strain associated with severe reproductive failures, including widespread abortion events and high mortality rates in newborn piglets, commonly referred to as “abortion storms” in sow herds [26–28]. First identified in the USA in 2014, this lineage has since been reported in other regions, such as Peru (2015) [29], China (2017) [30], Korea (2022) [31], and Japan (2022) [32], often presenting clinical manifestations similar to those observed in the initial USA outbreaks. Consistently, our retrospective analysis of a NADC34-like PRRSV field outbreak in Korea over a 38-week period (including 19 weeks before and after the outbreak) revealed that while the virus did not significantly impact the weaned piglet population directly, it was associated with a high incidence of abortions and neonatal piglet mortality [33]. Of note, the virus also contributed to increased pregnant sow mortality [33]. In that report, affected sows exhibited severe systemic inflammatory lesions with multi-organ involvement, suggesting that systemic inflammatory injury may contribute to maternal deterioration and adverse fetal outcomes during late gestation.

Given the recent emergence of NADC34-like (L1A) PRRSV and its association with unusually severe reproductive disease, we aimed to define its pathogenic features in pregnant sows relative to the prevalent NADC30-like (L1C) PRRSV using a late-gestation sow challenge model. The novelty of this study lies in (i) a direct comparison of NADC34-like versus NADC30-like PRRSV under the same experimental conditions, and (ii) integrated assessment of clinical outcomes, vertical transmission patterns, and immunopathological signatures across maternal tissues, the MFI, and fetal compartments. These data provide mechanistic context for recent field observations and inform future strategies to mitigate reproductive losses caused by emerging PRRSV-2 variants.

## Materials and methods

### Animals

Yorkshire × Landrace crossbred grandparent (GP) sows (parity 3 ± 1) were obtained from a high-health status GP breeding farm. The farm was regularly monitored for the absence of PRRSV through serological (ELISA) and molecular (PCR) testing, and for PCV2, PEDV, pathogenic *Escherichia coli*, and *Actinobacillus pleuropneumoniae* (APP) through molecular (PCR) testing. Only clinically healthy, PRRSV-negative sows were selected for this study.

As part of standard farm management, gilts introduced from the great-grandparent (GGP) breeding farm were vaccinated against PCV2 (Ingelvac CircoFLEX®, Boehringer-Ingelheim) and *Mycoplasma hyopneumoniae* (Ingelvac MycoFLEX®, Boehringer-Ingelheim) upon arrival. At every post-farrowing, sows were routinely vaccinated with a commercial swine erysipelas vaccine mixed with a classical swine fever virus strain LOM (Green Cross Veterinary Products Co., Ltd., Korea). Other vaccines of porcine parvovirus (PPV; PRO-VAC® PPV, Komipharm Co., Ltd., Korea), foot-and-mouth disease virus (FMDV; Bioaftogen, Biogénesis Bagó SA, Argentina) were also routinely applied in week intervals.

Estrus in sows used for this experiment was induced through daily direct boar exposure starting 3–4 days post-weaning. Artificial insemination was conducted using homospermic semen from Duroc boars. Pregnancy was confirmed using ultrasonography, and pregnant sows were individually housed in gestation stalls until approximately gestation day 87 (± 1). At this point, animals were transferred to a biosafety level 2 (BSL2) animal care facility at the Korea Research Institute for Veterinary Biologics (KRIVB). Prior to transfer, blood samples were collected via the jugular vein for confirmation of PRRSV-negative status by ELISA (PRRS Ab ELISA 4.0, BioNote Inc., Republic of Korea) and PCR (Prime-Q PCV2/PRRSV Detection Kit, Genet Bio, Republic of Korea). Upon arrival at the BSL2 facility, pregnant sows were randomly assigned to one of three experimental groups: JBNU-22-N01 (*n* = 4), PJ73 (*n* = 4), and a negative control (NC) group (*n* = 4). Each experimental group was housed separately, and sows were maintained in individual crates. Throughout the experimental period in the BSL2 facility, all sows had ad libitum access to water and were provided a standard wheat/barley-based gestation diet totaling 5 kg per day, divided into three feedings.

### PRRSV isolates

Two PRRSV-2 isolates were utilized in this study: JBNU-22-N01 (NADC34-like PRRSV, PRRSLoom classification 1A.29; GenBank Accession No. OP970983) and JB15-N-PJ73-GN (NADC30-like PRRSV, PRRSLoom classification 1C-unclassified; hereafter referred to as PJ73; GenBank Accession No. MZ287317). Virus classification was determined using the PRRSLoom variant classification system [34, 35]. These isolates were selected on the basis of previously described genetic characteristics [31, 36] and pathogenic profiles [37], as demonstrated in our earlier experimental infection studies using weaned piglet models.

Both PRRSV isolates were propagated in primary porcine alveolar macrophage (PAM) cells derived from PRRSV-negative, 4-week-old piglets maintained under controlled laboratory conditions, as described previously [31]. PAM cells were cultured in Gibco® RPMI-1640 medium (Life Technologies, USA) supplemented with 10% heat-inactivated fetal bovine serum (FBS), 2 mM L-glutamine, and 1 × Anti-Anti solution (100 IU/mL penicillin, 100 μg/mL streptomycin, and 0.25 μg/mL amphotericin B; Life Technologies). Cells were incubated at 37 °C in a humidified atmosphere containing 5% CO_2_. Viral titers were quantified by observing cytopathic effects (CPE) and reported as median tissue culture infectious dose per milliliter (TCID_50_/mL), following established protocols [38]. Virus stocks were aliquoted and stored at −80 °C. Immediately before inoculation, one aliquot of each viral isolate was thawed and diluted in phosphate-buffered saline (PBS) to achieve a final concentration of 1 × 10^4^ TCID_50_ in a total volume of 4 mL.

### Experimental procedures

The animal experimental protocol was reviewed and approved by the Institutional Animal Care and Use Committee (IACUC) of the Korea Research Institute for Veterinary Biologics (KRIVB) (approval number: KVD-2024-001) and conducted according to the guidelines and regulations outlined by the committee.

After a 9-day acclimation period in the BSL2 facility, pregnant sows were inoculated at gestation day 96 ± 1 (0 days post-challenge, dpc). JBNU-22-N01 (*n* = 4) and PJ73 (*n* = 4) groups were administered 1 × 10^4^ TCID_50_ of the respective PRRSV isolate in a total volume of 4 mL, via intramuscular injection (IM; 2 mL) using a sterile disposable syringe with 21-gauge needles and intranasal instillation (IN; 1 mL per nostril) using mucosal atomization devices (MAD Nasal™, Teleflex, USA). The dual-route inoculation protocol was adopted following established reproductive PRRSV challenge models [39, 40]. NC sows (*n* = 4) were similarly mock-inoculated with PBS. Daily monitoring included rectal temperature measurements and assessment of feed intake.

At 8 dpc (gestation day 104 ± 1), half of the sows from each group were humanely euthanized, and comprehensive necropsies were performed on both sows and their fetuses. The gravid reproductive tract was carefully removed, rinsed of maternal blood, and opened linearly starting from the tip of each uterine horn. Fetuses were sequentially numbered according to their position within each horn, with “L1” and “R1” designating those closest to the left and right ovaries, respectively. The preservation status of each fetus was categorized as viable, meconium-stained, decomposed (dead with primarily white skin), or autolyzed (dead with over 50% brown discolored skin), according to previously established criteria [41]. The weights of all fetuses at 8 dpc were recorded, and the size were measured with crown-to-rump length. The remaining sows were maintained until farrowing (approximately 18 − 20 dpc; gestation day 115 ± 1) to evaluate reproductive outcomes. The weights and sizes of live-born or aborted fetuses were also documented at farrowing. After farrowing, remaining sows were humanely euthanized and necropsied at 25 dpc.

### Sample collection

Serum, nasal swab, and rectal swab samples were collected from sows on days 0, 5, 8, 12, 15, and 25 dpc, aliquoted, and stored at −80 °C. At each necropsy (8 and 25 dpc), tissue samples of tonsil, thymus, lung, heart, liver, spleen, kidney, ovary, mandibular lymph node (LN), inguinal LN, and tracheobronchial LN were collected from each sow. The collected tissue samples were immediately frozen at −80 °C for subsequent viral load quantification and cytokine measurement or fixed in 10% neutral-buffered formalin (NBF) for microscopic evaluation.

At 8 dpc, amniotic fluid was collected from the amniotic cavity prior to fetal extraction. Umbilical cord blood was sampled from each fetus, aliquoted, and stored at −80 °C. Additionally, fetal tissues including brain, thymus, lung, heart, liver, spleen, kidney, umbilical cord, and associated endometrium were collected and immediately frozen at −80 °C. At farrowing, peritoneal fluid was aspirated from aborted fetuses and stillborn piglets, aliquoted, and stored at −80 °C for subsequent viral load quantification.

### Anti-PRRSV-specific IgG ELISA

Serum samples of 0, 5, 8, 12, 15, and 25 dpc were tested for anti-PRRSV IgG antibodies using a commercial ELISA kit (PRRS Ab ELISA 4.0; BioNote Inc., Republic of Korea) following the manufacturer’s instructions. Samples with an S/P ratio > 0.4 were considered positive for PRRSV antibodies.

### Quantification of PRRSV viral load

For tissue samples, 0.1 g of tissue was minced, diluted in phosphate-buffered saline (PBS) (1:10), and homogenized using a Beadbeater TissueLyser II (QIAGEN Inc., Germany). Viral RNA was extracted from 200 μL of tissue homogenates or fluidic samples (including serum, nasal swabs, rectal swabs, amniotic fluid, umbilical cord blood, and peritoneal fluid) using HiQ Viral DNA/RNA kit (BioD, Korea) with a NanoPrep32 Nucleic acid Extractor (BioD, Korea) according to the manufacturer’s instructions. Reverse transcription quantitative PCR (RT-qPCR) was performed using the Prime-Q PCV2/PRRSV Detection Kit (Genet Bio, Republic of Korea) on a CFX96 Real-Time PCR Detection System (Bio-Rad, USA) to quantify PRRSV viral loads.

To determine PRRSV genomic RNA copy numbers in each sample, a 1231-bp PCR fragment covering the ORF5–ORF6 region of PRRSV-2, which contains the target sequence of the qPCR kit, was amplified using the PrimeScript™ One-step RT-PCR Kit (Takara Bio, Japan). The reaction was conducted following the manufacturer’s instructions with the forward primer (11947-F: 5’-GGTGGGCAACTGTTTTAGCCT-3’) and the reverse primer (13729-R: 5’-GGCACAGCTGATTGACTGGC-3’). The PCR products were cloned into the pGEM-T Easy Vector (Promega, USA) to generate standard curves based on serial tenfold dilutions of the plasmid constructs. The absolute quantification of PRRSV genomic RNA was performed by normalizing the qPCR results to the standard curve (Additional file 1). Absolute PRRSV copy numbers were calculated from the standard curve. Viral loads were calculated from the standard curve and expressed as log_10_ genome copies per 200 μL of extraction input (tissue homogenate or fluid sample), accounting for the fixed RNA input per reaction and the total RNA elution volume.

To obtain an interpretable measure of multi-tissue fetal viral burden, tissue-specific viral loads were back-transformed to the non-log scale and summed across fetal tissues, followed by log_10_ transformation with a +1 pseudocount. The calculation of the outcome measure, termed Fetal Viral Burden (Fetal VB) was performed as follows:

$$Fetal VB = {\mathrm{log}}_{10}(\sum_{i=1}^{n}{10}^{{v}_{i}}+1)$$

where ${v}_{i}$ represents the log_10_-transformed viral load in tissue $i$, and $n$ is the number of tissues analyzed.

### Cytokine immunoassay from maternal and fetal tissues

From each sow, 0.5 g of tissue was minced and placed in a Safe-Lock microtube containing a sterile steel bead. Approximately 1 mL of T-PER^TM^ Tissue Protein Extraction Buffer, supplemented with Halt^TM^ Protease and Phosphatase Inhibitor Cocktail (Thermo Fisher Scientific, USA), was added to each tube and incubated for 5 min at room temperature. The tissues were then homogenized using a TissueLyser II (QIAGEN, Germany). The same procedure was applied to fetal lung, umbilical cord, and endometrial tissues. The homogenates were centrifuged at 13 000 rpm for 10 min at 4 °C, and the protein concentration of the resulting supernatant was measured using the Pierce^TM^ BCA Protein Assay Kit (Thermo Fisher Scientific, USA), following the manufacturer’s instructions. All protein samples were diluted to 5 mg/mL with T-PER^TM^ buffer and used for cytokine immunoassay.

The cytokine levels (IFN-α, IFN-γ, IL-1β, IL-4, IL-6, IL-8, IL-10, TNF-α, and IL-12p40) in tissue homogenates were measured using a ProcartaPlex^TM^ Porcine Cytokine and Chemokine Panel 1 9-Plex Immunoassay (Thermo Fisher Scientific, USA), according to the manufacturer’s instructions. The concentration of each cytokine was determined using a Luminex® 200 system (Luminex Corporation, USA). Cytokine concentrations were expressed as pg/mL of homogenate supernatant.

### Microscopic assessment of PRRSV-associated lesions

At each necropsy (8 and 25 dpc), tissue samples from sows including lung, mandibular LN, inguinal LN, liver, aorta, kidney, and heart were fixed in 10% neutral-buffered formalin (NBF), embedded in paraffin, and sectioned at 3–4 μm thickness. Sections were stained with hematoxylin and eosin (H&E) for histopathological examination.

Microscopic findings of PRRSV-associated lesions were assessed in the lungs, lymph nodes, blood vessels, heart, and liver on the basis of the criteria as previously described [42]. The severity and distribution of lesions in each tissue section scored on a five-point scale from 0 (absent) to 4 (severe/widespread) based on both severity and distribution using light microscope (Olympus, Japan) following Krakowka et al. [43]. Lung-specific interstitial pneumonia was scored according to Guo et al. [44], and mononuclear inflammatory cell infiltrate assessment followed the criteria of Malgarin et al. [45]. For group-level comparison, individual lesion scores across evaluated tissues were also summed for each sow to provide a composite index of overall histopathological burden.

To detect PRRSV antigens in various organs, immunohistochemistry (IHC) was performed on the basis of a previously described protocol [46], with minor modifications. Dako REAL^TM^ EnVision^TM^ Detection System, Peroxidase/DAB + Rabbit/Mouse (K 5007, Dako, Denmark) was used for IHC according to the manufacturer’s instructions. The tissue sections were placed on silane-coated slides (MUTO PURE CHEMICALS, Japan), deparaffinized and rehydrated. They were quenched with 3% hydrogen peroxide (H_2_O_2_) in PBS to remove endogenous peroxidase and digested with 0.05% protease XIV (Sigma, USA) for 10 min at 37 °C for antigen retrieval. After washing in PBS, Anti-PRRSV Monoclonal Antibody (9041, Median Diagnostics, Republic of Korea) diluted 1:1,000 in Antibody diluent solution (Dako, USA) was added for 60 min at 37 °C in a humidified chamber. The sections were washed with PBS and treated with Dako REAL EnVision/HRP, Rabbit/Mouse (Dako, Denmark) for 40 min at 37 °C in a humidified chamber. The reaction was visualized using applying 3, 3’-diamino-benzidine tetrahydrochloride, DAB chromogen (Dako, Denmark), and then, the tissue sections were counterstained with Mayer’s hematoxylin.

### Total RNA extraction and mRNA quantification from endometrium

Total RNA was extracted from 0.1 g of endometrial tissues using 0.5 mL of NucleoZOL® reagent and the NucleoSpin® RNA Set for NucleoZOL (Macherey–Nagel, Germany), according to the manufacturer’s instructions. The extracted RNA was confirmed using a NanoDrop spectrophotometer (Biospec-nano, Shimadzu Scientific Instruments, Japan), followed by Qubit 2.0 fluorometer using a Qubit RNA Broad Range (BR) assay kit for quantification. Subsequently, 5 μg of RNA was used for complementary DNA (cDNA) synthesis using a WizScript^TM^ cDNA Synthesis Kit (Wizbiosolutions, Republic of Korea), according to the manufacturer’s instructions.

To elucidate disruptions in TJ integrity in endometrium due to PRRSV-2 infection, *CLDN1*, *CLDN4*, *CLDN5*, *CLDN6*, *CLDN10*, and *TJP1* were selected as target genes, as previously described [20, 23, 47, 48]. Additionally, *CDH1* gene (encoding E-cadherin), a key component of adherens junctions (AJs) that maintains epithelial cell adhesion [21], and *CXADR* (coxsackievirus and adenovirus receptor) gene, a member of the JAM family essential for AJ and TJ assembly [49], were included to further assess epithelial barrier integrity. To evaluate immune regulation and inflammatory responses, immune checkpoint molecules (*PD1* and *PDL1*), interferon-stimulated genes (ISGs; *ISG15* and *ISG12[A]*), and M2-macrophage differentiation markers (*SPP1* and *TREM2*), previously investigated in PRRSV-infected bronchoalveolar lavage (BAL) cells, were analyzed to assess their involvement in PRRSV-induced immunomodulation within the endometrium.

Quantitative real-time PCR (qPCR) was performed on a 7500 Fast Real-time PCR system (Applied Biosystems, USA) with a Power SYBR^TM^ Green PCR master mix kit (Applied Biosystems, USA). The comparative cycle threshold (Ct) method (2^−ΔΔCt^) was used for relative quantification by normalizing target genes to the expression of the housekeeping gene porcine *HPRT1* [50]. All qPCR results were confirmed by melting curve analysis, and the primer set sequences for the target and housekeeping genes are provided in Additional file 2.

### Statistics and data visualization

All statistical analyses and data visualization were performed using GraphPad Prism (version 10.0.3; GraphPad Software) or R software. Sow body temperature, feed intake, viral loads, histopathological scores, cytokine concentrations, and gene expression levels were analyzed using two-way analysis of variance (ANOVA) followed by Tukey’s multiple comparison test. Fetal weights and sizes at 8 and 25 dpc were analyzed by one-way ANOVA with Tukey’s post hoc test, while the sizes of aborted fetuses at 25 dpc were compared using a nonparametric Mann–Whitney *U* test, as comparisons were limited to JBNU-22-N01 and PJ73 groups. Pearson correlation analyses were conducted using GraphPad Prism for scatter plot regression analyses, and Spearman correlation matrices were generated in R using the “corrplot” package, with significance defined at *p* < 0.01. Principal component analysis (PCA) was performed using the prcomp() function in R to visualize and classify fetuses and associated MFI tissues, including the umbilical cord and endometrium, based on their viral loads.

For heatmap visualization of cytokine concentrations and gene expression levels, all data were log_2_-transformed, and robust Z-scores were calculated to normalize across samples and reduce the influence of outliers. Heatmaps were generated using the pheatmap package in R. For maternal tissue cytokines, samples were organized by infection group (strain) and tissue type, without hierarchical clustering. For datasets from fetal compartments and the maternal–fetal interface (endometrium, umbilical cord, and fetal lungs), samples were manually ordered on the basis of infection group, viral load cluster assignment, and endometrial viral load ranking.

To assess the independent effects of PRRSV strain and viral load on cytokine responses and gene expression at the maternal–fetal interface, multiple linear regression analyses were conducted. Cytokine concentrations from the endometrium, umbilical cord, and fetal lungs, and gene expression levels from the endometrium, including tight junction-associated genes (*CLDN1, CLDN4, CLDN5, CLDN6, CLDN10, TJP1*), adherens junction components (*CDH1*, *CXADR*), immune checkpoint molecules (*PD1*, *PDL1*), interferon-stimulated genes (*ISG15, ISG12[A]*), and macrophage markers (*TREM2*, *SPP1*), were modeled with PRRSV strain (categorical: JBNU-22-N01, PJ73, and negative control) and log-transformed viral load as independent variables. Least-squares regression models were fitted in GraphPad Prism, and the significance of each predictor was determined by ANOVA. Model fit was evaluated using R-squared values, and multicollinearity was assessed via variance inflation factors (VIF), with VIF values below 10 considered acceptable. Residual normality was assessed using the Shapiro–Wilk test. For cytokine analyses, residuals did not always meet normality assumptions, which is common in biological datasets due to inherent variability. Nonetheless, regression models were retained as they demonstrated clear and biologically consistent patterns, with robust regression coefficients and significant predictor effects. Gene expression analyses satisfied all normality assumptions. For interpretation, outcomes were classified as strain-dependent when the strain effect (ANOVA) showed *p* < 0.01, and as absolute viral load-dependent when the viral load effect (ANOVA) showed *p* < 0.01.

### Sample selection and RNA library construction

Among a total of 70 porcine endometrial samples, nine representative samples (*n* = 3 per group) were selected for transcriptome analysis based on two criteria applied jointly: (i) PRRSV RNA load in the endometrium, with samples chosen to span the observed range of viral burden within each experimental group; and (ii) expression levels of key junction-associated genes assessed by targeted qPCR, ensuring that selected samples were representative of the group-level expression patterns rather than biological outliers. The groups comprised L1A_MVL (JBNU-22-N01; Lineage 1A with medium viral load), L1C_MVL (PJ73; Lineage 1C with medium viral load), and NEG (PRRSV-negative controls). Total RNA was extracted, and sample quality was assessed using the Agilent 2100 Bioanalyzer (Agilent Technologies). Only samples with an RNA integrity number (RIN) exceeding 7.0 were used for sequencing. RNA libraries were prepared using the TruSeq Stranded mRNA Library Prep Kit (Illumina) and sequenced on the Illumina NovaSeq 6000 platform to generate 150 bp paired-end reads.

### RNA-seq data processing and differential gene expression analysis

Raw reads underwent adapter trimming and quality filtering using Trimmomatic [51] and FastQC. Cleaned reads were aligned to the *Sus scrofa* reference genome (Sscrofa11.1, Ensembl v113) using HISAT2 [52]. Gene-wise abundances of each sample were calculated on the basis of the exons annotated in the Ensembl Sscrofa11.1 GTF file (release 113), using the FeatureCounts software [53], and subsequently filtered to exclude genes with fewer than 10 counts across all samples. Normalization and statistical modeling were performed using the edgeR [54] package of Bioconductor [55]. Normalization of the raw counts was performed using the trimmed mean of M-value (TMM) method, and dispersion parameters were estimated and applied using the Cox–Reid profile-adjusted likelihood method in edgeR [54]. Differential expression between groups was assessed using a quasi-likelihood F-test (glmQLFTest), controlling for overdispersion. Genes with a false discovery rate (FDR) < 0.05 and absolute log_2_ fold-change (log_2_FC) > 2 were defined as differentially expressed genes (DEGs). Pairwise comparisons were performed as follows: C1 (NEG versus L1C_MVL), C2 (NEG versus L1A_MVL), and C3 (L1C_MVL versus L1A_MVL). Multidimensional scaling (MDS) was performed using the limma [56] function of the R package to identify the similarities among samples.

### Functional enrichment and network analysis

Gene Ontology (GO) and Kyoto Encyclopedia of Genes and Genomes (KEGG) pathway enrichment analyses were conducted using the clusterProfiler [57] package in R. Both over-representation analysis (ORA) and gene set enrichment analysis (GSEA) [58] were applied to differentially expressed genes (DEGs) filtered by FDR < 0.05 and |log_2_FC|> 2. GSEA was performed using ranked gene lists based on the product of logFC × −log_10_(FDR), with GO analysis restricted to the Biological Process (BP) ontology and KEGG analysis performed using *Sus scrofa* annotations (organism code: ssc).

For KEGG GSEA results, emapplot function was employed to visualize the interactions and semantic similarity network of enriched pathways. From this network, a distinct cluster of seven immune-related KEGG pathways was identified. Genes within these pathways were extracted, and their differential expression values (log_2_FC) were compared between L1A_MVL and L1C_MVL groups, each relative to the NEG group.

### Validation of RNA-seq data with qPCR

To validate the RNA-seq findings, quantitative real-time PCR (qPCR) was performed on selected genes showing the most pronounced differences in expression between the two PRRSV-infected groups. Genes were chosen on the basis of their known roles in antiviral immunity and their upregulation in previous studies of PRRSV infection, including SPP1, CXCL10, MX1. The gene expression levels of pan-T cell marker CD2 and complement gene *C1R* were also evaluated. Total RNA was extracted from the same endometrial tissue samples used for sequencing, and cDNA synthesis was carried out using the WizScript^TM^ cDNA Synthesis Kit (Wizbiosolutions, Republic of Korea). qPCR was conducted using the Power SYBR^TM^ Green PCR Master Mix (Applied Biosystems, USA) on a 7500 Fast Real-Time PCR System (Applied Biosystems, USA). Relative gene expression was calculated using the 2^−ΔΔCt^ method with *HPRT1* gene as the internal control. Primer sequences for the target and reference genes are listed in Additional file 2.

### Measurement of serum kynurenine/tryptophan ratio

To evaluate whether systemic immunoregulatory mechanisms involving the kynurenine pathway contribute to immune modulation during PRRSV-2 infection, we quantified serum concentrations of kynurenine (Kyn) and tryptophan (Trp) in sows from all experimental groups. This analysis was prompted by the observation that the JBNU-22-N01 (L1A) group exhibited elevated pathogenicity and robust IFN-α responses, yet displayed reduced immune-related gene expression in the endometrium compared with the PJ73 (L1C) group. Serum samples of sows collected at 0, 5, 8, 12, 15, and 25 dpc were analyzed using a kynurenine/tryptophan ratio ELISA kit (Cat. No. ISE-2227; ImmuSmol, France), following the manufacturer’s protocol. Optical densities were measured at 450 nm using a microplate reader, and Kyn and Trp concentrations were calculated from standard curves.

## Results

### Clinical signs and litter outcomes

**Figure 1:**
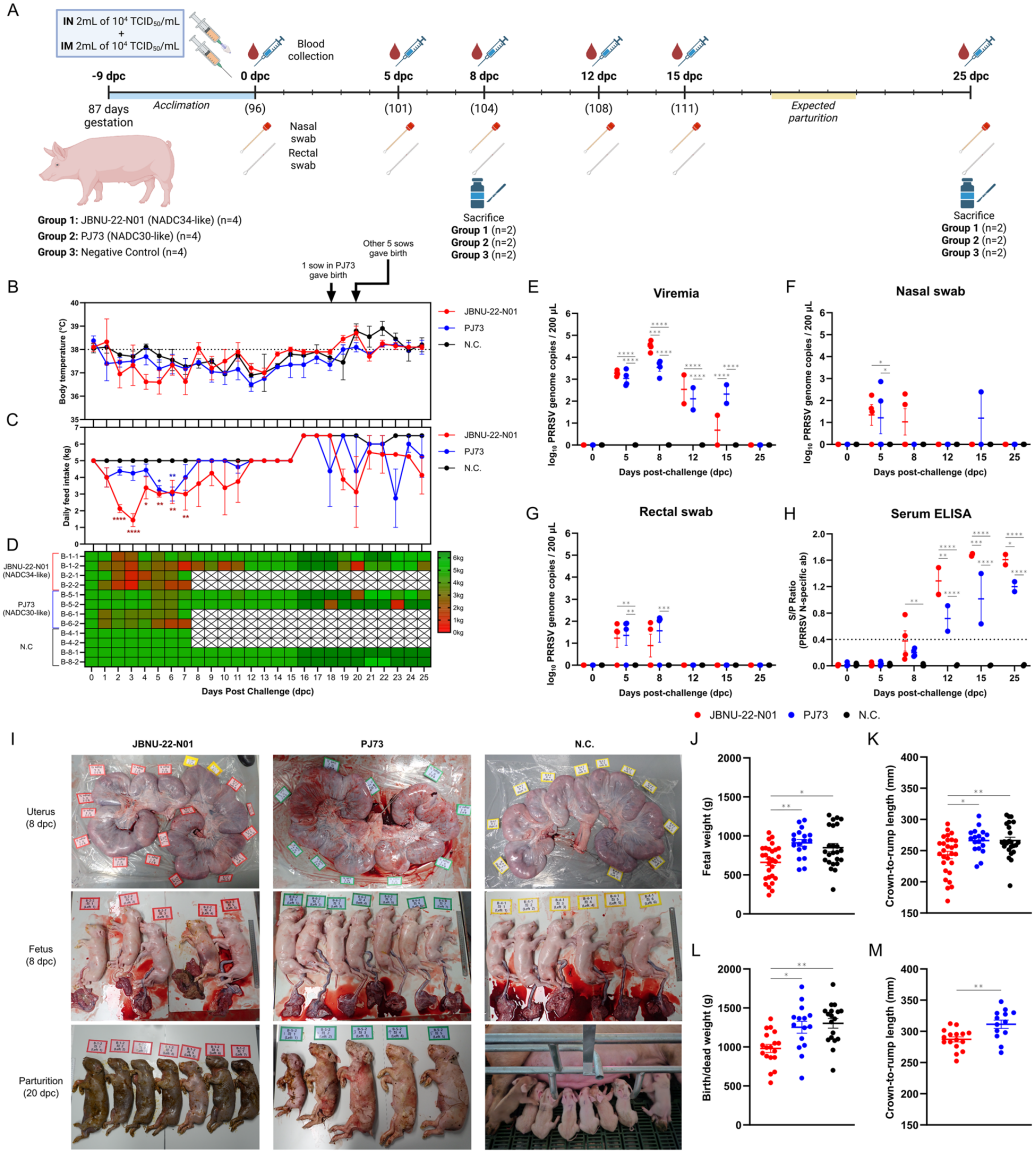
**Experiment design and clinical outcomes in PRRSV-infected pregnant sows**. **A** Schematic overview of the experimental timeline. **B** Daily rectal temperature measurements. **C**–**D** Daily feed intake. (E–G) PRRSV RNA quantification in serum **E**, nasal swabs **F**, and rectal swabs **G** across different time points. **H** Serum PRRSV-specific IgG levels measured by ELISA. **I** Fetal preservation status at necropsy or farrowing. **J**–**M** Fetal weights and sizes at 8 dpc (**J**, **K**) and parturition (**L**, **M**). Data are presented as mean ± SEM. Statistical significance: *p < 0.05, **p < 0.01, ***p < 0.001, ****p < 0.0001.

During the experimental period (Figure 1A), no pregnant sow exhibited respiratory signs, including dyspnea, persistent paroxysmal coughing, or lethargy, following PRRSV infection. Additionally, no sows displayed signs of depression or prolonged inappetence. Fever, defined as a rectal temperature exceeding 39.5 °C at any point after PRRSV inoculation, was observed in only one sow from the JBNU-22-N01-infected group at 1 dpc (Figure 1B). Reduced daily feed intake was the most pronounced clinical sign among PRRSV-infected sows (Figure 1C and D), with JBNU-22-N01-infected sows exhibiting significantly reduced feed intake from 2 to 7 dpc. In contrast, PJ73-infected sows showed a significant reduction in feed intake only between 5 and 6 dpc, suggesting a difference in pathogenicity between the two strains.

PRRSV serum viremia was significantly higher in the JBNU-22-N01 group compared with the PJ73 group at 8 dpc (*p* < 0.001), whereas PJ73-infected sows exhibited significantly higher viremia at 15 dpc (*p* < 0.0001) (Figure 1E). However, other forms of viral shedding, including nasal viral load (Figure 1F) and rectal viral load (Figure 1G), did not show significant differences between the two infection groups. The serum PRRSV non-neutralizing antibody response, which typically reflects the magnitude of serum viral load [59], was detected earlier in the JBNU-22-N01-infected group, with significant antibody levels compared with negative control (*p* < 0.01) observed from 8 dpc onward. This group consistently exhibited significantly higher antibody titers than the PJ73 group throughout the experimental period (Figure 1H). These findings suggest differences in viral replication kinetics between the JBNU-22-N01 and PJ73 strains.

The weights and sizes of fetuses were measured at 8 dpc and at parturition, following the experimental design (Figure 1A and I). At 8 dpc, a total of 28 fetuses from the JBNU-22-N01 group, 18 fetuses from the PJ73 group, and 24 fetuses from the NC group were collected via necropsy, with each group comprising fetuses from two sows. The summary of fetal preservation is illustrated in Figure 4A. Fetuses from JBNU-22-N01-infected sows exhibited significantly lower weights and sizes (*p* < 0.05) than those from PJ73-infected or NC sows (Figure 1J and K), suggesting more severe intrauterine growth restriction (IUGR).

At parturition, a total of 18 fetuses were obtained from each group (JBNU-22-N01, PJ73, and NC) from two sows, respectively. Although two fetuses (11.1%, 2/18) in both the JBNU-22-N01 and PJ73 groups were born alive but weak, all of these piglets died within 2 days post-parturition. Consistent with the findings at 8 dpc, the JBNU-22-N01 group exhibited significantly lower weights and sizes compared with the PJ73 and NC groups (Figure 1L and M). The consistent reduction in fetal body weight and size at both time points suggests that JBNU-22-N01 infection resulted in an earlier and more severe impairment of fetal growth compared with PJ73 infection.

Additionally, viral load measurements from the peritoneal fluid of dead-born piglets showed high PRRSV RNA levels in both the JBNU-22-N01 and PJ73 groups, but the difference was not statistically significant ( Additional file 3).

### Viral loads and cytokine levels in sow tissues

**Figure 2:**
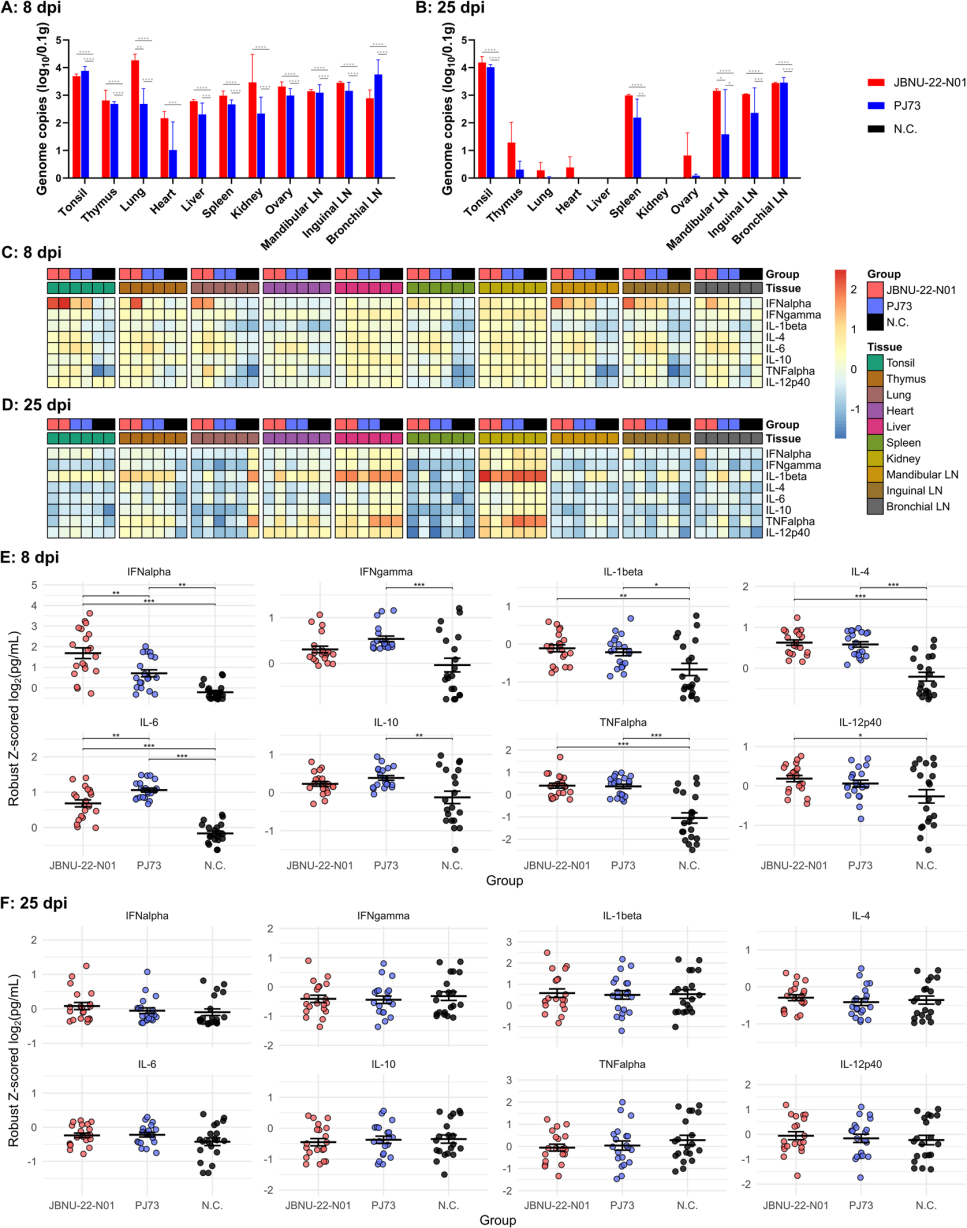
**PRRSV RNA loads and cytokine profiles in maternal tissues of infected pregnant sows.** (A–B) PRRSV RNA viral loads in maternal tissues (tonsil, thymus, lung, heart, liver, spleen, kidney, mandibular lymph node, inguinal lymph node, bronchial lymph node) at **A** 8 dpc and **B** 25 dpc. **C**–**D** Heatmaps depicting Z-scored log_2_(pg/mL) cytokine concentrations (interferon (IFN)-α, IFN-γ, interleukin (IL)-1β, IL-4, IL-6, IL-10, tumor necrosis factor (TNF)-α, IL-12p40) across maternal tissues at **C** 8 dpc and **D** 25 dpc. **E**–**F** Aggregate cytokine levels (Z-scored log_2_(pg/mL)) pooled across all tissues per infection group at **E** 8 dpc and **F** 25 dpc. Data are presented as mean ± SEM. Statistical significance: *p < 0.05, **p < 0.01, ***p < 0.001, ****p < 0.0001.

To evaluate the tissue distribution of PRRSV, viral RNA loads were quantified in multiple maternal tissues, including tonsil, thymus, lung, heart, liver, spleen, kidney, mandibular lymph node (LN), inguinal LN, and bronchial LN, at 8 days post-challenge (dpc) and 25 dpc (Figure 2A and B). At 8 dpc, coinciding with peak serum viremia (Figure 1E), JBNU-22-N01-infected sows (NADC34-like PRRSV) exhibited significantly higher viral loads in the lung (*p* < 0.01) compared with PJ73-infected sows (NADC30-like PRRSV). Although not statistically significant, viral loads in the heart and kidney of JBNU-22-N01-infected sows tended to be higher than those of PJ73-infected sows. By 25 dpc, viral RNA was largely cleared from most tissues except for lymphoid tissues (tonsils, spleen, lymph nodes), indicating persistent viral reservoirs.

Cytokine concentrations (IFN-α, IFN-γ, IL-1β, IL-4, IL-6, IL-10, TNF-α, IL-12p40) were measured in the same tissues at 8 and 25 dpc to assess local immune responses (Figure 2C and D). Heatmap analyses revealed tissue-specific cytokine expression patterns. At 8 dpc, IFN-α levels, a type I interferon known to rise in parallel with viral load during early PRRSV infection [26], were notably elevated in tissues of tonsil, thymus, lung, mandibular, and inguinal lymph nodes of JBNU-22-N01-infected sows (Additional file 4). However, other cytokines displayed variable, tissue-dependent patterns across both infection groups.

To discern broader strain-dependent cytokine trends, cytokine levels were aggregated across all tissues for each infection group (Figure 2E and F). At 8 dpc, JBNU-22-N01-infected sows exhibited higher systemic levels of IFN-α and IL-12p40, indicating a stronger type I interferon and proinflammatory response. Conversely, PJ73-infected sows showed elevated levels of IFN-γ, IL-6, and IL-10, suggesting a more pronounced adaptive and regulatory cytokine profile. By 25 dpc, overall cytokine levels diminished in both groups, though strain-dependent differences remained discernible.

### Microscopic findings of PRRSV-associated lesions in sow tissues

**Figure 3:**
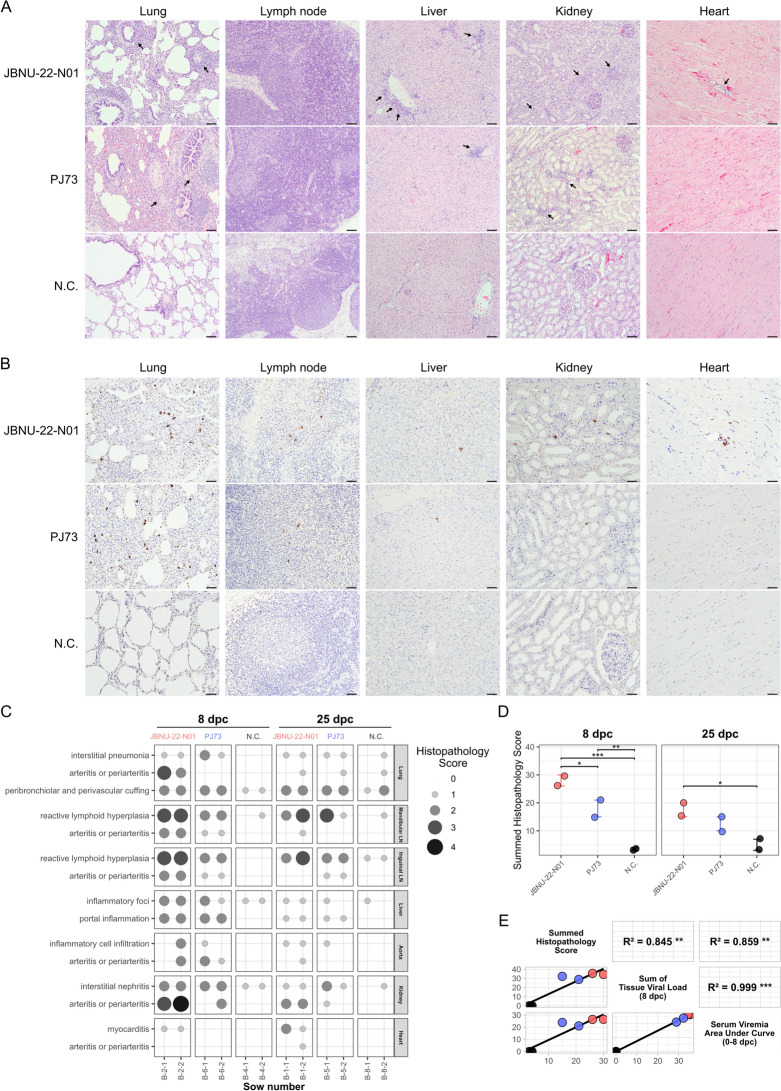
**Histopathological evaluation and PRRSV antigen detection in sow tissues.** **A** Representative H&E-stained micrographs of lung, LNs, liver, kidney, and heart tissues from each group at 8 dpc. JBNU-22-N01-infected sows showed more extensive inflammatory infiltrates and vascular lesions compared with PJ73 and NC sows. Scale bars = 100 μm. **B** Immunohistochemical detection of PRRSV antigen in the same tissues using monoclonal antibody 4A5. Antigen-positive cells (brown staining) were predominantly in the lung, LNs, and kidney of infected sows, particularly in JBNU-22-N01-infected animals. No PRRSV-positive staining was observed in NC sows. Scale bars = 50 μm. **C** Bubble plot summarizing histopathological scores from lung, LNs, liver, kidney, and heart tissues. Lesions were graded on a scale from 0 (absent) to 4 (severe) for each sow and tissue, based on H&E-stained sections. **D** Summed histopathology scores by sow and group at 8 dpc and 25 dpc. Data are presented as mean ± SEM. Statistical significance: *p < 0.05, **p < 0.01, ***p < 0.001, ****p < 0.0001. **E** Correlation analyses between summed histopathological scores, summed tissue viral load at 8 dpc, and serum viremia area under the curve (AUC) from 0 to 8 dpc.

Histopathological evaluation was performed to assess the severity and distribution of PRRSV-associated lesions in sow tissues at 8 dpc and 25 dpc, expressed as histopathological scores (HPS) (Figure 3). At 8 dpc, the most frequently observed histopathological lesions included arteritis or periarteritis in various organs (lung, lymph nodes (LNs), liver, kidney, aorta, and heart) and reactive lymphoid hyperplasia in the mandibular and inguinal LNs (Figure 3A and C; see Additional file 5). At 25 dpc, the severity of vascular inflammatory lesions, including arteritis/periarteritis, was generally reduced in both infection groups compared with 8 dpc. However, reactive lymphoid hyperplasia and some residual lesions in the lung and LNs persisted from 8 to 25 dpc (Figure 3C). Interestingly, although mild-to-moderate interstitial pneumonia with peribronchiolar and perivascular cuffing was noted in some animals (Additional file 5A and B), it was not a prominent feature across all infected animals (Figure 3C), despite being a hallmark pathological finding in PRRSV-infected weaned piglet models [5], suggesting that PRRSV-associated pathology in pregnant sows may differ from that in younger pigs, potentially reflecting variations in immune response or disease progression across age groups.

Immunohistochemical analysis at 8 dpc showed that specific positive reaction for PRRSV antigens was detected in lung, LNs, liver, and kidney in both JBNU-22-N01-infected sows. However, no positive staining was detected in the heart of one JBNU-22-N01-infected sow. In the PJ73 infection group, a specific positive reaction for PRRSV antigens were also detected in lung, LNs, and kidney in both sows. Negative reaction was showed in one liver and both hearts in PJ73-infected sows. In the NC group, No PRRSV antigen was detected in all organs. According to the IHC result, viral antigens were distributed in a broader range of internal organs in JBNU-22-N01-infected sows than PJ73-infected sows at the early stage of infection.

To provide an overall summary of lesion burden across organs, individual HPS values were also summed for each sow into a total HPS score, while lesion-specific patterns were interpreted separately on the basis of tissue distribution and lesion type. When individual HPS values were summed into a total HPS score, both JBNU-22-N01-infected and PJ73-infected sows exhibited significantly higher summed HPS compared with the NC group (*p* < 0.01), with JBNU-22-N01-infected sows displaying a trend toward higher scores than PJ73-infected sows (Figure 3D). By 25 dpc, lesion severity was markedly reduced in all infection groups, although lymphoid hyperplasia persisted in PRRSV-infected sows. Notably, JBNU-22-N01-infected sows continued to exhibit a higher summed HPS compared with other groups (Figure 3B), suggesting prolonged or more severe histopathological changes.

A strong positive correlation was observed between summed tissue viral load and viremia (calculated as area under the curve, AUC) (*R*^2^ = 0.9989, *p* < 0.0001) (Figure 3E). Additionally, the summed HPS at 8 dpc was strongly correlated with both summed tissue viral load (*p* < 0.01) and viremia AUC (*p* < 0.01) (Figure 3E), consistent with an association between viral replication and tissue lesion burden.

### Fetal outcomes and tissue viral loads

**Figure 4:**
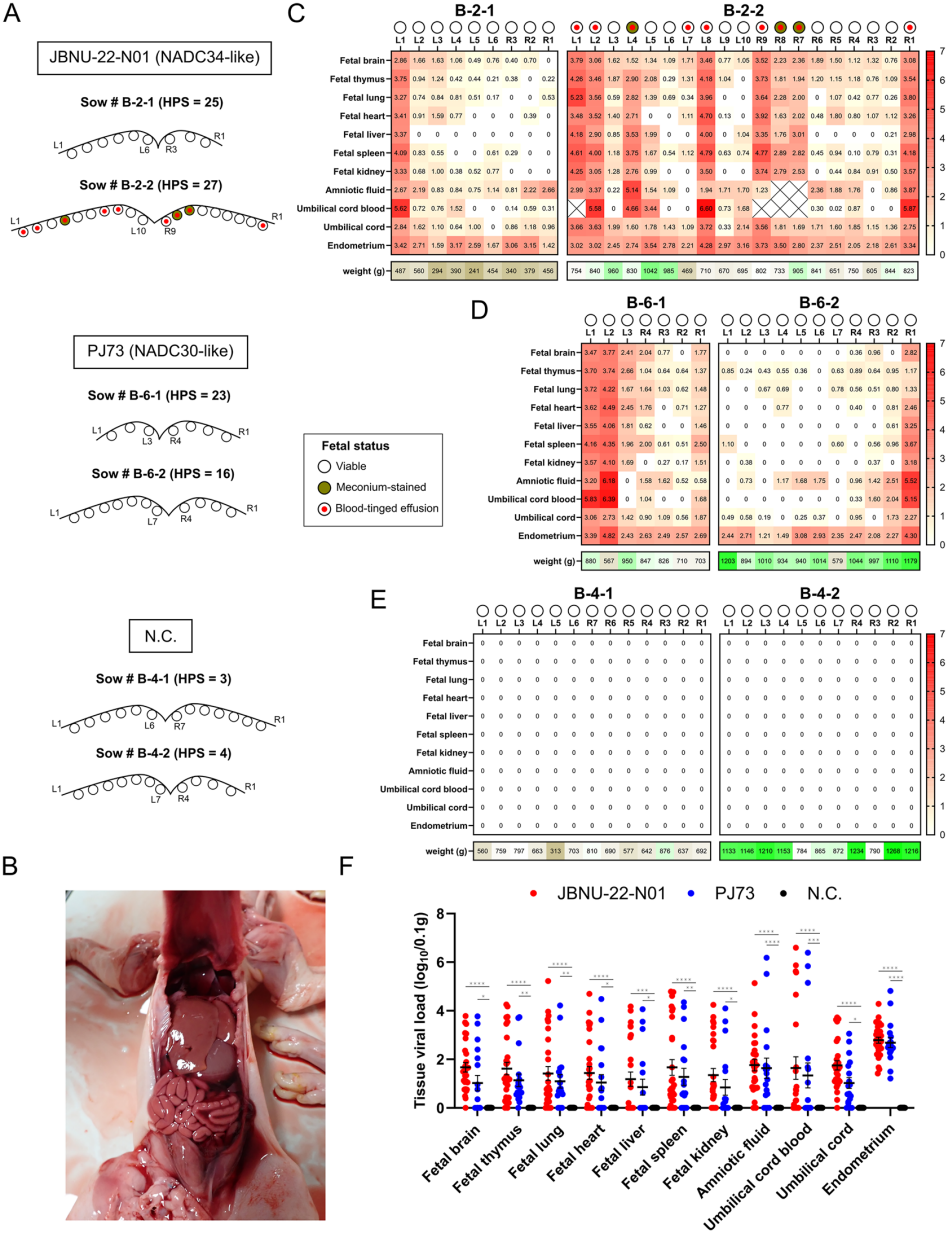
**Fetal preservation status and PRRSV tissue distribution in fetuses.** **A** Fetal preservation status at 8 dpc, categorized as viable or meconium-stained. JBNU-22-N01-infected fetuses exhibited a higher frequency of meconium staining. **B** Representative images of fetal blood-tinged effusion frequently observed in JBNU-22-N01-infected fetuses. **C** PRRSV RNA loads in various fetal tissues, umbilical cord blood, amniotic fluid, and associated endometrial tissues at 8 dpc. PRRSV RNA was detected in all examined fetal-associated endometrial tissues and multiple fetal tissues, with no significant difference between JBNU-22-N01 and PJ73 groups. Data are presented as mean ± SEM. Statistical significance: *p < 0.05, **p < 0.01, ***p < 0.001, ****p < 0.0001.

The litter outcomes from all six pregnant sows at 8 dpc (JBNU-22-N01; *n* = 2, PJ73; *n* = 2, and NC; *n* = 2) are summarized in Figure 4A, including fetal numbers, locations, and preservation status. Given that PRRSV-induced fetal compromise typically emerges from 8 dpc and worsens through 14 dpc, fetuses at 8 dpc displayed only two preservation states: viable and meconium-stained. Notably, only three fetuses (10.7%, 3/28) in the JBNU-22-N01-infected group exhibited meconium staining (Figure 4C), whereas none were observed in the PJ73-infected (Figure 4D) or NC groups (Figure 4E). Interestingly, blood-tinged effusion was frequently observed during tissue collection and dissection, affecting 32.1% (9/28) of JBNU-22-N01-infected fetuses, including those with meconium staining (Figure 4B), suggesting a potential association between PRRSV infection, endothelial disruption, and fetal vascular instability, potentially mediated by direct viral effects or associated immune responses.

Across both JBNU-22-N01- and PJ73-infected groups, all fetal-associated endometrial tissues tested PRRSV-positive, with significant variability in viral loads within individual sows depending on specific endometrial location. Similarly, differences in viral presence and magnitude were observed across fetal tissues, with some exhibiting high viral loads, while others in the same sow showed low or undetectable levels. This heterogeneous viral distribution across fetuses within the same sow is consistent with previous reports [60]. Notably, viral loads in all fetal tissues showed significant positive correlations, indicating a synchronized viral spread within the fetus (Additional file 6). A similar correlation was observed between fetal tissues and their associated endometrial samples, suggesting that local viral burden in the endometrium directly influences fetal infection dynamics. While JBNU-22-N01-infected fetuses exhibited a trend toward higher viral loads in both fetal tissues and the endometrium compared with PJ73-infected fetuses, the difference was not statistically significant (Figure 4F), likely due to inter-fetal variability and localized factors.

### Correlations of viral loads in fetal compartments and MFI-fetus groupings

**Figure 5:**
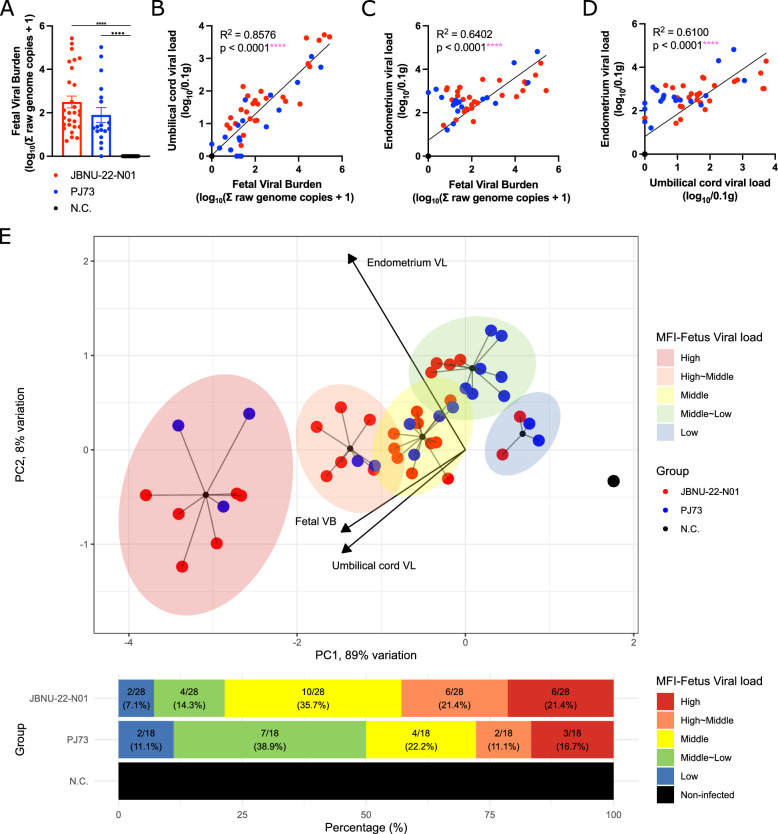
**Correlation between viral load and fetal–maternal interface (MFI) dynamics. A** Summed fetal viral load (Fetal VL SUM) across different fetal tissues. **B**–**D** Correlation analysis between Fetal VL SUM, umbilical cord viral load, and associated endometrial viral load. Strong correlations were observed between fetal viral burden and maternal–fetal transmission. **E** Principal component analysis (PCA)-based classification of fetuses into six viral load groups. JBNU-22-N01-infected fetuses exhibited a higher frequency of high viral load clusters compared with PJ73-infected fetuses. Statistical comparison between groups was performed using Fisher’s exact test (OR = 1.95, *p* = 0.361). Data are presented as mean ± SEM. Statistical significance: *p < 0.05, **p < 0.01, ***p < 0.001, ****p < 0.0001.

To provide a single fetus-level summary of infection across multiple sampled tissues, we calculated Fetal Viral Burden (Fetal VB) as log_10_(Σ raw viral genome copies across fetal tissues +1). This composite measure was used to capture overall fetal viral burden while avoiding direct summation of log_10_-transformed values (Figure 5A). Viral loads measured across individual fetal tissues showed strong positive correlations with each other, consistent with coordinated multi-organ infection patterns (Additional file 6). In addition, Fetal VB was strongly associated with viral loads in the umbilical cord (*R*^2^ = 0.8576, *p* < 0.0001) (Figure 5B) and in the endometrium (*R*^2^ = 0.6402, *p* < 0.0001) (Figure 5C). A significant correlation was also observed between umbilical cord and endometrial viral loads (*R*^2^ = 0.6100, *p* < 0.0001) (Figure 5D), further reinforcing the concept that maternal–fetal viral transmission occurs primarily through umbilical circulation [60].

To facilitate better interpretation and further analysis, fetuses were clustered into six viral load groups (High, High-Middle, Middle, Middle-Low, Low, and Non-infected) using principal component analysis (PCA) and k-means clustering (Figure 5E). These groups were intended as a data-driven descriptive framework for visualization and downstream interpretation, rather than as fixed biological classes or predefined pathological thresholds. Notably, the JBNU-22-N01-infected group exhibited a higher frequency of fetuses in viral load categories with greater PRRSV burden compared with the PJ73-infected group. Specifically, in the JBNU-22-N01 group, 42.9% (12/28) of fetuses fell into the High or High-Middle viral load clusters, whereas 27.8% (5/18) of fetuses in the PJ73 group were categorized in these higher viral load clusters (Figure 5E). These findings suggest that JBNU-22-N01 may exhibit enhanced fetal tropism or greater efficiency in maternal–fetal transmission compared with PJ73. Although this difference in cluster distribution did not reach statistical significance (Fisher’s exact test, OR = 1.95, *p* = 0.361), likely reflecting the limited number of fetuses per group, the observed pattern is directionally consistent with the overall trend of greater fetal viral burden in JBNU-22-N01-infected animals (Figures 4E and 5A).

### Gene expression and cytokine landscapes across the MFI and fetal compartments

**Figure 6:**
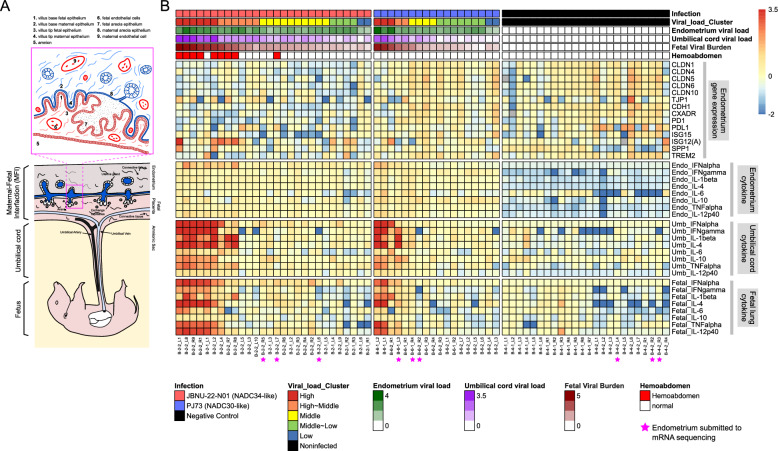
**Spatial distribution of gene expression and cytokine responses across the maternal–fetal interface (MFI) and fetal compartments.** **A** Schematic representation of the MFI, umbilical cord, and fetal compartments, illustrating the histological structures and spatial relationships between maternal endometrium, placental tissues, umbilical vasculature, and the fetus. **B** Heatmaps depicting gene expression levels in the endometrium (top) and cytokine concentrations across the endometrium, umbilical cord, and fetal lungs (bottom). Gene expression data were log_2_ fold change (–ΔΔCt) values normalized using robust Z-scores, while cytokine concentrations (pg/mL) were log_2_-transformed and normalized with robust Z-scores to enable comparative visualization across tissues. Samples were manually ordered on the basis of infection group (strain), viral load cluster, and endometrial viral load, as shown by the annotation panels at the top of each heatmap. These panels indicate infection group (color-coded), viral load classification (High, High-Middle, Middle, etc.), and relative viral loads in the endometrium, umbilical cord, and fetus, facilitating visualization of patterns associated with PRRSV strain and fetal viral burden across maternal and fetal compartments. Samples marked with pink stars indicate those selected for subsequent mRNA sequencing (NGS) analysis as representative subsamples from each viral load group and infection category.

To elucidate the spatial distribution of immune and structural responses along the maternal–fetal axis, we visualized gene expression profiles in the endometrium and cytokine responses across the maternal–fetal interface (MFI), umbilical cord, and fetal lungs (Figure 6). The histological schematic (Figure 6A) illustrates the complex architecture of the MFI and its connections to the umbilical circulation and fetal compartments, providing anatomical context for interpreting gene expression and cytokine data.

**Figure 7:**
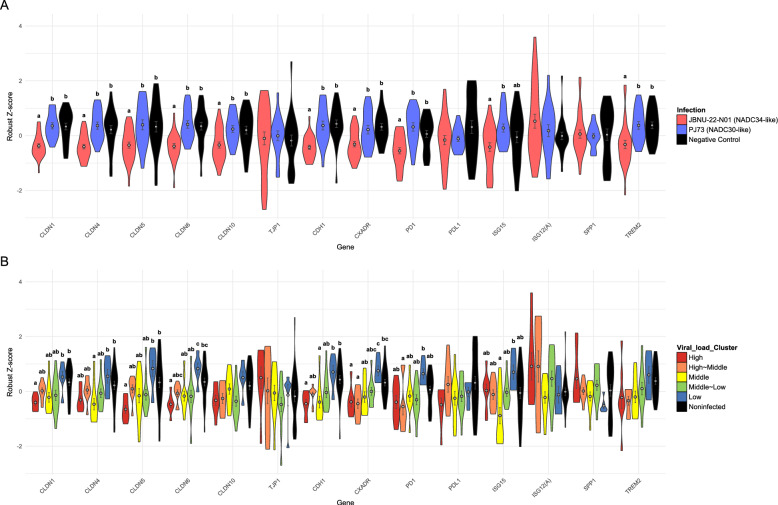
**Strain- and viral load-wise modulation of gene expression in endometrium.** Gene expression levels of cell junction-related genes (tight junction-associated: *CLDN1, CLDN4, CLDN5, CLDN6, CLDN10, TJP1*; adherens junction components: *CDH1*, *CXADR*) and immune-related genes (*PD1, PDL1, ISG15, ISG12[A], SPP1, TREM2*) in the endometrium were normalized using robust Z-scores applied to the log_2_ fold change (–ΔΔCt) values. **A** Gene expression patterns stratified by infection group (strain-wise) comparison. **B** Gene expression patterns stratified by fetal viral load clusters (viral load-wise), irrespective of strain. Statistical significance among groups was assessed using two-way analysis of variance (ANOVA) with Tukey’s multiple comparison test, and denoted by different letters above each bar; bars not sharing a letter are significantly different (*p* < 0.05).

Heatmap visualization of endometrial gene expression (Figure 6B, top) revealed distinct expression patterns of cell junction-associated genes (tight and adherens junction components) and immune-related genes, stratified by infection group, viral load cluster, and endometrial viral load. Notably, downregulation of tight junction genes (*CLDN1, CLDN4, CLDN5, CLDN6*, and *CLDN10*) and adherens junction components (*CDH1* and *CXADR*) was more pronounced in JBNU-22-N01-infected sows, particularly within high viral load clusters. To further quantify these differences, gene expression levels were compared between infection groups (strain-wise) and viral load clusters (viral load-wise) (Figure 7). Strain-wise comparisons showed significant downregulation of multiple junction-associated genes in JBNU-22-N01 infection compared with PJ73 and control groups, whereas viral load-wise analyses revealed more modest or inconsistent associations with absolute viral burden.

**Figure 8:**
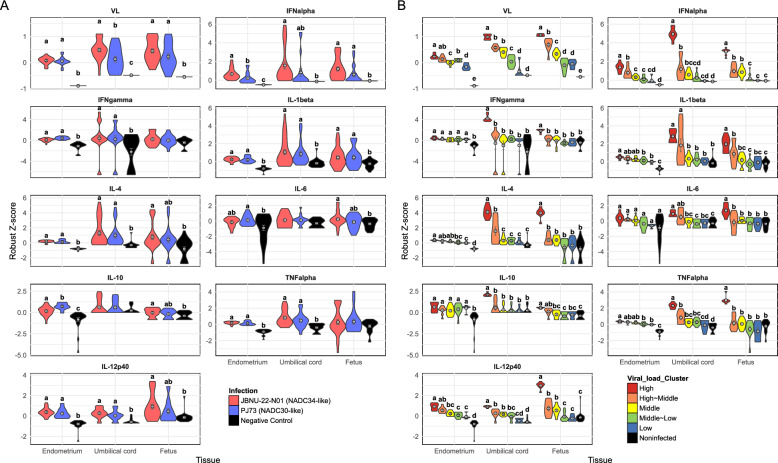
**Strain- and viral load-wise cytokine responses across the maternal–fetal interface and fetal compartments.** Cytokine concentrations (pg/mL) measured in the endometrium, umbilical cord, and fetal lungs were log_2_-transformed and normalized using robust Z-scores for visualization. **A** Cytokine expression patterns stratified by infection group (strain-wise) comparison. **B** Cytokine expression patterns stratified by fetal viral load clusters (viral load-wise), irrespective of strain. Statistical significance among groups was assessed using two-way ANOVA with Tukey’s multiple comparison test, and indicated by different letters above each bar; bars not sharing a letter are significantly different (*p* < 0.05).

In contrast, cytokine responses exhibited tissue-specific distribution patterns (Figure 6B, bottom). While endometrial cytokines displayed a broad distribution with moderate associations to both strain and viral load clusters, umbilical cord and fetal lung cytokine levels were tightly aligned with fetal viral load clusters, with proinflammatory cytokines (IFN-α, IFN-γ, IL-1β, TNF-α and IL-12p40), as well as antiinflammatory cytokines (IL-4 and IL-10), markedly elevated in fetuses with higher viral burdens (Figure 8). These patterns suggested a divergence in regulatory mechanisms between maternal and fetal immune environments.

To further investigate the independent contributions of PRRSV strain and viral load to these observed patterns, multiple linear regression (MLR) analyses were performed for both gene expression (Additional file 7) and cytokine concentrations (Additional file 8). Regression models confirmed that junction-associated gene expression in the endometrium was predominantly strain-dependent, with JBNU-22-N01 infection exerting stronger downregulatory effects than PJ73, irrespective of viral load. For immune-related genes, including *PD1, PDL1, ISG15*, and *TREM2*, both strain and viral load influenced expression, although strain effects remained dominant in most cases. Such strain-dependent disruption of tight junction-associated gene expression likely compromises placental barrier integrity, promoting maternal–fetal viral transmission [20, 23]. This hypothesis is supported by the higher proportion of fetuses in elevated viral load clusters in JBNU-22-N01 infection (Figure 5), indicating that epithelial barrier weakening at the maternal–fetal interface may facilitate greater fetal exposure to PRRSV, contributing to adverse outcomes.

In contrast, cytokine responses across the MFI and fetal compartments exhibited distinct patterns of regulation. MLR analyses showed that endometrial cytokine responses (e.g., IFN-γ, IL-10) were primarily strain-dependent, whereas cytokine levels in the umbilical cord and fetal lungs (e.g., TNF-α, IL-12p40) were predominantly viral load-dependent (Additional file 8). These findings corroborated the visual patterns observed in Figures 6 and 8, emphasizing that maternal immune responses are more influenced by viral strain characteristics, while fetal immune responses are driven by the absolute viral burden, which were aligned with previous findings that cytokine-related gene expression in fetal tissues is more accurately predicted by fetal viral load [16, 17, 24].

### Transcriptomic profiling of the endometrium reveals strain-specific immunomodulation

**Figure 9:**
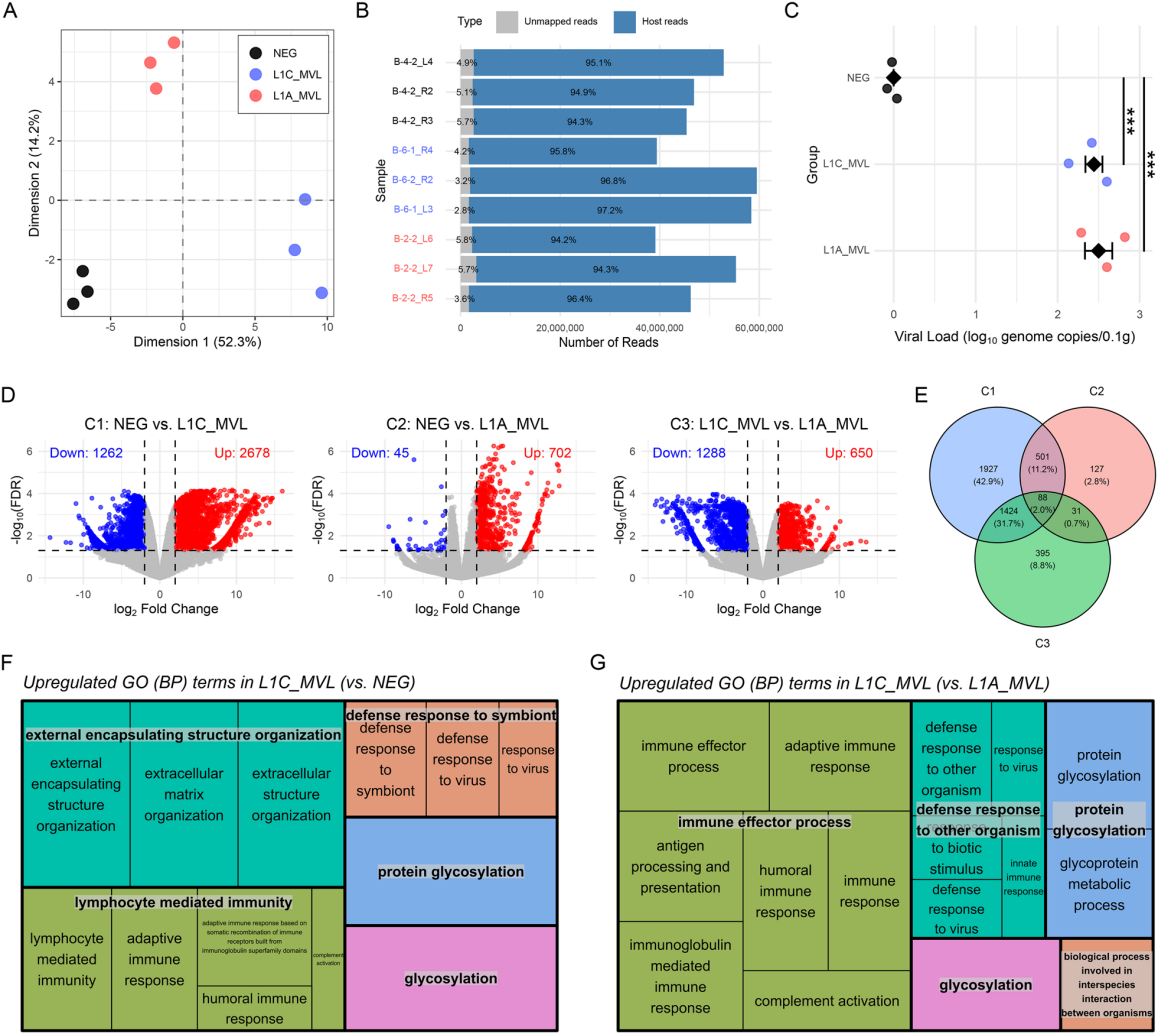
**Transcriptomic profiling of endometrial tissues.** **A** Multidimensional scaling (MDS) plot showing distinct transcriptional clustering of endometrial samples by infection group: NEG (black), L1C_MVL (blue), and L1A_MVL (red). **B** Summary of RNA-seq data quality for each sample, showing total number of reads and mapping rates. **C** Endometrial PRRSV viral load. **D** Volcano plots displaying differentially expressed genes (DEGs) for each comparison (C1: NEG versus L1C_MVL; C2: NEG versus L1A_MVL; C3: L1C_MVL versus L1A_MVL) using FDR < 0.05 and |log_2_FC|> 2. **E** Venn diagram illustrating DEG overlap among comparisons. (F–G) REVIGO treemaps of upregulated Gene Ontology Biological Process (GO:BP) terms enriched in L1C_MVL versus **F** NEG and **G** L1A_MVL. Only terms significant in both over-representation analysis (ORA) and gene set enrichment analysis (GSEA) are displayed.

To explore local host responses at the maternal–fetal interface, transcriptomic profiling was performed using endometrial tissues collected at 8 dpc from sows infected with JBNU-22-N01 (L1A_MVL) or PJ73 (L1C_MVL), as well as uninfected controls (NEG). A total of nine representative samples (*n* = 3 per group) were selected on the basis of viral load cluster assignment and sample quality. All RNA-seq libraries showed high mapping rates (> 94%) with over 40 million clean reads per sample (Figure 9B), and endometrial PRRSV viral loads were tightly matched between L1A_MVL and L1C_MVL groups (Figure 9C). This design allowed for comparison of host responses with PRRSV infection while minimizing viral load as a confounding variable.

MDS analysis revealed clear transcriptional separation among the three groups, with L1C_MVL and L1A_MVL samples clustering distinctly from NEG controls and from each other (Figure 9A). A total of 3941 DEGs were identified across three pairwise comparisons, with 3930 DEGs in L1C_MVL versus NEG (C1), 747 DEGs in L1A_MVL versus NEG (C2), and 1938 DEGs in L1C_MVL versus L1A_MVL (C3), using an FDR threshold of < 0.05 and |log_2_FC|> 2 (Figure 9D). The Venn diagram highlights both shared infection-responsive genes and lineage-skewed sets (Figure 9E), with L1C_MVL exhibiting a more upregulated term than L1A_MVL ( Additional file 9). Consistently, REVIGO treemaps of upregulated GO:BP terms show immune/inflammatory processes enriched in L1C_MVL when contrasted with NEG and with L1A_MVL (Figure 9F and G).

### Functional enrichment and immune module characterization

**Figure 10:**
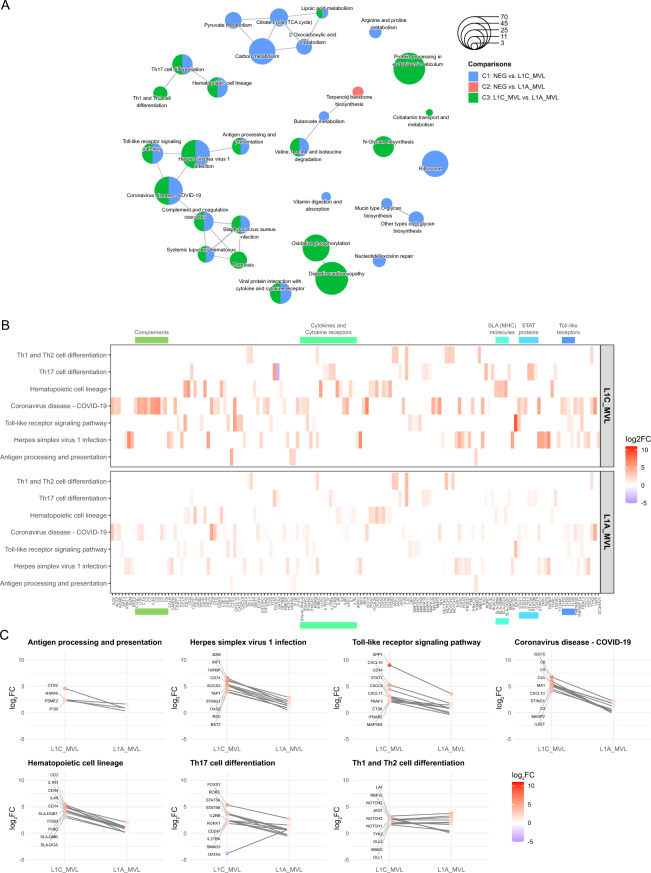
**Differential immune gene expression across PRRSV-infected endometrial samples**. **A** Network representation of GSEA-enriched KEGG pathways on the basis of semantic similarity clustering. **B** Heatmap of log_2_ fold-change values for genes within the seven selected immune-related KEGG pathways across L1C_MVL and L1A_MVL groups, each compared with NEG. Immune effector genes, including MHC molecules, cytokines, and TLRs, show robust upregulation in L1C_MVL and attenuated expression in L1A_MVL. **C** Paired slope plots showing gene-specific log_2_FC comparisons between L1C_MVL and L1A_MVL across pathways.

Similarly, KEGG-based ORA and GSEA revealed a strong activation of immune-associated pathways in L1C_MVL samples, including Th1 and Th2 cell differentiation, Toll-like receptor signaling pathway, hematopoietic cell lineage, and Herpes simplex virus 1 infection, compared with both NEG and L1A_MVL. By contrast, L1A_MVL samples demonstrated little to no enrichment in these pathways, with only one significantly downregulated KEGG pathway, terpenoid backbone biosynthesis, meeting the significance threshold (Additional file 10). To functionally characterize key immune modules, significantly enriched KEGG pathways from GSEA were visualized as a network on the basis of semantic similarity. A distinct immune module comprising seven pathways was identified, enriched in both the C1 (NEG versus L1C_MVL) and C3 (L1C_MVL versus L1A_MVL) comparisons (Figure 10A). Heatmap visualization revealed marked upregulation of immune effector genes in the L1C_MVL group, including complement components, SLA (MHC) molecules, Toll-like receptors, STAT signaling molecules, and various cytokines and their receptors. In contrast, the same genes in L1A_MVL samples exhibited consistently attenuated expression or were not significantly differentially expressed (FDR > 0.05) compared with NEG (Figure 10B). Paired slope plots of representative pathways further emphasized this disparity, indicating a strain-dependent reduction in immune gene induction in response to JBNU-22-N01 infection (Figure 10C).

### Validation with qPCR

**Figure 11:**
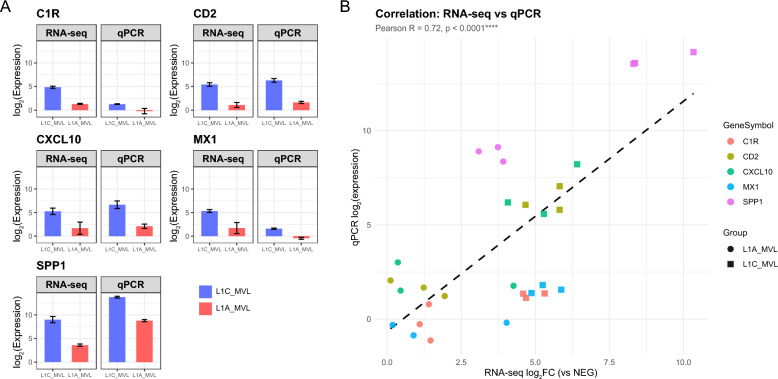
**qPCR validation.** **A** Bar plots comparing RNA-seq and qPCR expression levels for five representative immune-related genes: *C1R, CD2, CXCL10, MX1*, and *SPP1*. **B** Correlation between RNA-seq and qPCR measurements across all genes tested (Pearson R = 0.72, *p* < 0.0001). Each point represents the log_2_FC expression of a gene measured by both methods.

To validate the functional enrichment results at the transcript level, we examined the expression profiles of genes within the seven KEGG immune pathways across the L1C_MVL and L1A_MVL groups, each relative to NEG. qPCR was performed for five representative immune-related genes: *C1R* (complement), *CD2* (T cell marker), *CXCL10* and *MX1* (interferon-stimulated genes), and SPP1 (macrophage activation marker). Expression patterns assessed by qPCR were consistent with RNA-seq results, demonstrating significantly higher expression in L1C_MVL samples compared with L1A_MVL (Figure 11A). Furthermore, correlation analysis across all qPCR and RNA-seq measurements yielded a strong positive correlation (Pearson *R* = 0.72, *p* < 0.0001), supporting the reliability of the RNA-seq data (Figure 11B).

Together, these findings confirm that while both PRRSV strains achieved comparable endometrial viral loads, they elicited distinct host transcriptional programs. The PJ73 strain (L1C_MVL) triggered broad immune activation at the maternal–fetal interface, whereas the JBNU-22-N01 strain (L1A_MVL) was associated with attenuated immune gene expression, particularly within innate recognition and T cell–related modules.

### Potential involvement of kynurenine pathway in immunoregulatory responses

Given the observation that JBNU-22-N01 infection was associated with elevated IFN-α levels and yet a paradoxically dampened endometrial immune gene expression compared with PJ73, we investigated whether systemic immunoregulatory pathways may have contributed to this discrepancy. We focused on the kynurenine pathway, an IFN-inducible metabolic cascade known to mediate immunosuppression via the catabolism of tryptophan into kynurenine.

**Figure 12:**
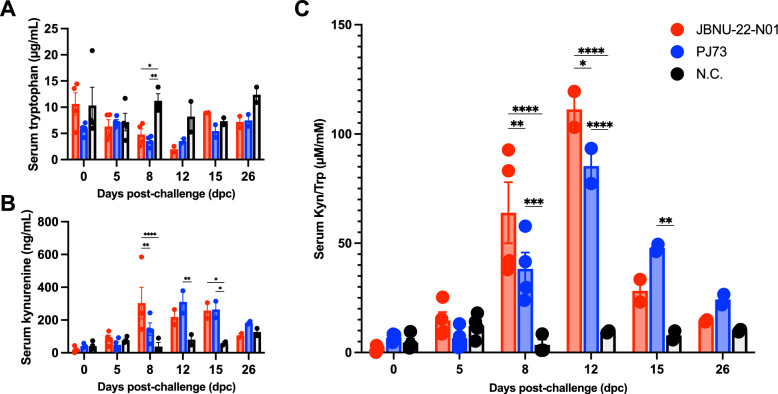
**Temporal dynamics of serum tryptophan, kynurenine, and kynurenine/tryptophan (Kyn/Trp) ratio in sows following PRRSV challenge.** Serum concentrations of tryptophan (top left), kynurenine (bottom left), and the calculated Kyn/Trp ratio (right) were measured at multiple time points (0, 5, 8, 12, 15, and 25 dpc) in sows infected with JBNU-22-N01 (red), PJ73 (blue), or mock-inoculated (N.C., black). Data are presented as mean ± SEM. Statistical significance: *p < 0.05, **p < 0.01, ***p < 0.001, ****p < 0.0001.

Serum levels of tryptophan and kynurenine were longitudinally measured throughout the post-infection period, and the kynurenine/tryptophan (Kyn/Trp) ratio was calculated as a surrogate marker of indoleamine 2,3-dioxygenase (IDO) activity. At 8 and 12 dpc, JBNU-22-N01-infected sows exhibited significantly elevated serum kynurenine concentrations (*p* < 0.0001), while serum tryptophan was markedly reduced (*p* < 0.05), leading to a pronounced increase in Kyn/Trp ratio (*p* < 0.0001) compared with both PJ73-infected and control animals (Figure 12). Although PJ73 infection also led to moderate increases in kynurenine levels, the Kyn/Trp ratio remained significantly lower than that observed in JBNU-22-N01 infection at peak response time points.

## Discussion

Previous studies on NADC34-like PRRSV have predominantly focused on weaned piglet models, yielding variable outcomes ranging from mild virulence [61–64] to severe [28], leaving its overall pathogenicity inconclusive. In our recent study, we conducted a comparative pathogenicity analysis of JBNU-22-N01, PJ73, and the PRRSV-2 prototype strain VR2332 in weaned piglets, incorporating comprehensive immunological assessments [65]. JBNU-22-N01 infection induced typical PRRSV-associated clinical signs such as interstitial pneumonia and reduced weight gain, but did not result in mortality, unlike highly virulent PRRSV strains including HP-PRRSV or PRRSV-1 Lena strain [26]. These findings categorized JBNU-22-N01 as moderately pathogenic or nonlethal in the weaned piglet model. However, beyond clinical manifestations, JBNU-22-N01 demonstrated enhanced replication kinetics in in vitro study [31], along with a robust proinflammatory cytokine response followed by increased immunosuppressive effects, including elevated IL-10 and immune checkpoint molecules (PD-1, CTLA-4, IDO1) compared with other strains [65]. These immunomodulatory properties, together with the enhanced viral replication kinetics, suggest that both viral replication and immune dysregulation may synergistically contribute to the clinical severity observed in pregnant sows and neonatal piglets. In addition to its association with severe reproductive failure, a recent field study reported an elevated pregnant sow mortality rate (~10%) during a NADC34-like PRRSV outbreak [33], implying that the virus could exert direct pathogenic effects on pregnant sows. Motivated by these findings, the present study aimed to characterize the pathogenic features of JBNU-22-N01 in pregnant sows during late gestation, with a particular focus on immunopathological responses at both the maternal side and the MFI, as well as associated maternal–fetal viral transmission.

As both NADC34-like and NADC30-like PRRSV have been globally prevailing recently, several studies have examined their pathogenicity in pregnant sow models during late gestation, using field isolates including natural recombinant viruses derived from these lineages [66–68]. Although these studies reported that both lineages exhibited high virulence, resulting in elevated body temperature, reduced feed intake, and abortion, none directly compared the pathogenicity of these two distinct lineages in parallel, leaving their relative differences unclear. In this study, JBNU-22-N01 and PJ73 infections revealed clear distinctions in pathogenicity. JBNU-22-N01-infected sows exhibited more severe clinical manifestations, including significantly reduced feed intake and earlier, higher peak viremia during the acute phase, compared with PJ73-infected sows (Figure 1B–H). Furthermore, although maternal viral RNA loads were comparable between the two strains by 25 dpc, JBNU-22-N01 maintained a higher viral burden during the acute phase, particularly in the lung and lymphoid tissues (Figure 2A), reflecting its enhanced replication kinetics. Consistent with findings from the weaned piglet model [65], marked elevations of IFN-α, a type I interferon known to rise in parallel with viral load during PRRSV infection [26, 69], and proinflammatory cytokine IL-12p40 were observed across maternal tissues of JBNU-22-N01-infected sows at 8 dpc, indicating robust innate immune activation (Figs. 2D and E) [70, 71]. In contrast, PJ73 infection induced higher levels of adaptive and regulatory cytokines, including IFN-γ, IL-6, and IL-10, suggesting a more modulated immune response (Figure 2E) [70, 72, 73]. These immunological differences were mirrored in histopathological outcomes, inducing more severe lesions in JBNU-22-N01 infection including arteritis, periarteritis, and lymphoid hyperplasia, particularly at 8 dpc, with higher cumulative histopathological scores persisting until 25 dpc (Figure 3A and B). Such histopathological lesions associated with potential vascular inflammation was consistent with findings from field cases of NADC34-like PRRSV-associated pregnant sow mortalities [33]. These findings suggest that the heightened pathogenicity and clinical manifestations observed in pregnant sows infected with NADC34-like PRRSV may be influenced by both its enhanced replication kinetics and the accompanying systemic proinflammatory milieu, which could contribute to vascular inflammation. Similar to hypertensive disorders in human pregnancy, such as preeclampsia, which are characterized by vascular maladaptation and systemic inflammation [74], and may be exacerbated by virus-induced immune responses [75], the vascular lesions observed in JBNU-22-N01-infected sows raise the possibility of analogous pathogenic mechanisms. Although direct parallels between human and swine pregnancy remain speculative, the pronounced vascular pathology observed here highlights the potential vulnerability of the maternal cardiovascular system to PRRSV-induced inflammation. Future studies incorporating detailed hemodynamic, endothelial, and histological assessments will be essential to clarify the interplay between PRRSV infection, pregnancy-related vascular adaptations, and maternal disease severity in late gestation.

Following maternal infection, PRRSV vertical transmission represents a critical determinant of fetal outcome, particularly during late gestation when the placenta becomes more permeable to viral passage [2]. In this study, both JBNU-22-N01 and PJ73 were capable of inducing fetal infection, culminating in 100% reproductive failure at parturition following experimental challenge during late gestation, with all born piglets being nonviable (Additional file 3). However, the extent and severity of fetal viral burden differed markedly between the two strains. Despite comparable mean fetal viral loads between groups at 8 dpc (Figure 5A), fetuses from JBNU-22-N01-infected sows were disproportionately represented in high and high-middle viral load clusters, as revealed by PCA-based classification (Figure 5E). This pattern suggests a greater efficiency of transplacental viral transmission in JBNU-22-N01 infection, potentially owing to strain-specific features that enhance its tropism for or traversal across the maternal–fetal interface. Moreover, meconium staining and blood-tinged effusion, which are the hallmarks of fetal compromise and imminent death [2, 16, 24], were observed more frequently in JBNU-22-N01-infected fetuses (Figure 4), indicating that this strain not only increased the rate of fetal infection but also exerted a more severe intrauterine pathological impact. These findings support the hypothesis that viral burden alone does not fully account for fetal compromise. Instead, the interplay between viral replication dynamics, placental barrier disruption, and local immune responses likely acts in concert to shape fetal outcomes. In particular, the strong correlation observed between elevated endometrial viral loads and corresponding umbilical and fetal viral loads (Figure 5) highlights the pivotal role of localized maternal tissues at the MFI in facilitating viral dissemination and influencing fetal preservation status.

The spatial dynamics of viral transmission were further elucidated by examining gene expression and cytokine responses along the maternal–fetal interface (MFI). Specifically, JBNU-22-N01 infection was associated with significant downregulation of tight junction-related genes (*CLDN1, CLDN4, CLDN5*), particularly in high viral load clusters (Figures 6 and 7). These genes have previously been shown to play essential roles in maintaining epithelial barrier integrity at the MFI [20, 23], and their suppression may compromise placental architecture, thereby facilitating PRRSV passage into the fetal compartment. However, our data do not directly demonstrate placental detachment or a specific structural lesion caused by these transcriptional changes. Therefore, we interpret the observed downregulation of junction-associated genes as a plausible indicator of impaired maternal–fetal interface integrity rather than definitive evidence of placental separation. This aligns with earlier findings demonstrating that PRRSV-induced disruption of tight junctions correlates with increased fetal infection and impaired viability [23]. In addition, our study newly identified a marked downregulation of adherens junction-associated genes (*CDH1, CXADR*) in the JBNU-22-N01 infection group. While the role of tight junctions has been previously established, this observation suggests that adherens junction components may also contribute to maintaining endometrial epithelial integrity and could represent a novel mechanism by which PRRSV compromises the maternal–fetal barrier. Interestingly, these junctional alterations, both tight and adherens, were more strongly associated with viral strain than with absolute viral burden, as evidenced by multiple linear regression analyses (Additional file 7). This implies that specific viral determinants of the NADC34-like PRRSV may directly modulate epithelial structure and function, thereby contributing to the increased frequency of fetuses with high viral loads in JBNU-22-N01 infection, or alternatively, enabling more rapid transplacental viral transmission. This interpretation is further supported by the observation that JBNU-22-N01 infection was associated with higher endometrial viral loads, a greater proportion of fetuses in the high and high-middle viral burden groups, and more frequent signs of fetal compromise, including meconium staining and blood-tinged effusion. Together, these findings are consistent with the possibility that altered junctional integrity at the maternal side of the interface may facilitate viral dissemination and contribute indirectly to more severe fetal outcomes.

Cytokine responses along the maternal–fetal axis revealed striking compartmentalization, with maternal and fetal tissues exhibiting divergent regulatory patterns (Figure 6). In the endometrium, cytokine profiles were significantly influenced by both the PRRSV strain and the magnitude of local viral burden (Additional file 8), underscoring the combined effects of viral virulence factors and the local immune milieu. Notably, JBNU-22-N01 infection was associated with markedly elevated levels of IFN-α (Figure 8A), accompanied by strain-specific cytokine production patterns in other tissues (Figure 2E). In contrast, fetal compartments such as the umbilical cord and fetal lungs exhibited cytokine expression patterns that were predominantly shaped by absolute viral load, regardless of PRRSV strain (Figure 8B and Additional file 8). Proinflammatory cytokines, including TNF-α and IL-12p40, were significantly elevated in fetuses with higher viral burden, implicating these proinflammatory cytokines as indicators of active viral replication and tissue compromise. However, their expression levels did not differ significantly between strains, reinforcing the idea that elevated fetal cytokines reflect the downstream consequences of infection, such as tissue stress or impending demise, rather than being primary drivers of pathology [24]. Therefore, while cytokine profiling provides valuable insight into fetal immune status, its utility may lie more in serving as a biomarker of fetal viral exposure and disease progression than in identifying causative immunological events. This distinction is critical when interpreting immune activation in fetal compartments and underscores the need to focus investigative efforts on upstream maternal factors, including local immune responses in adjacent endometrium, that may drive transplacental virus transmission and irreversible fetal virus infection.

To investigate these upstream maternal mechanisms in greater detail, we performed transcriptomic profiling on selected endometrial samples from sows representing different infection groups but exhibiting similar viral burdens across the maternal–fetal axis (highlighted in Figure 6, pink stars). This subset allowed us to capture transcriptional signatures within the endometrium under distinct viral strain exposures while minimizing the confounding effect of absolute endometrial viral load (Figure 9C). Differential gene expression and pathway enrichment analyses revealed marked differences in endometrial responses between JBNU-22-N01 and PJ73 infections. While PJ73 infection triggered robust upregulation of antiviral and inflammatory pathways and genes, JBNU-22-N01 infection was characterized by a limited activation of immune pathways (Figures 9, 10, 11). These findings suggest that the NADC34-like PRRSV strain may preferentially antagonize immune activation, potentially facilitating viral evasion and enhancing transmission efficiency to the fetus. In contrast, the stronger activation of immune-related pathways observed in PJ73-infected sows may have contributed to a more effective antiviral environment at the MFI. This heightened immune vigilance may have delayed or limited the efficiency of transplacental virus transmission in PJ73 infection, thus providing a relative protective effect against fetal compromise despite comparable levels of maternal viral load.

To further explore systemic immunometabolic changes associated with the attenuated immune activation observed in JBNU-22-N01 infection, we measured serum kynurenine-to-tryptophan (Kyn/Trp) ratios. The Kyn/Trp ratio has often been used as an indirect indicator of altered tryptophan metabolism and may, under some conditions, reflect activation of the indoleamine 2,3-dioxygenase (IDO) pathway. IDO is classically induced by IFN-γ and can be further potentiated by other proinflammatory cytokines such as IL-1β and TNF-α [76, 77]. Once activated, this pathway can influence immune regulation by depleting tryptophan and increasing kynurenine metabolites, which have been linked to suppression of effector T-cell proliferation, promotion of regulatory T-cell differentiation, and macrophage polarization [78, 79]. The involvement of altered Kyn/Trp metabolism during PRRSV infection has also been reported in our weaned piglet model [80]. In the current study, sows infected with JBNU-22-N01 exhibited significantly elevated Kyn/Trp ratios compared with PJ73-infected counterparts (Figure 12). However, this finding should be interpreted cautiously, because the Kyn/Trp ratio is an indirect marker and may also be influenced by reduced feed intake, systemic inflammation, and other infection-associated metabolic changes. Therefore, in the absence of direct measurement of IDO1 expression or activity in relevant tissues or immune-cell compartments, the elevated Kyn/Trp ratio cannot be considered definitive evidence of IDO-mediated immunosuppression. Instead, we interpret this result as being consistent with altered tryptophan metabolism during more severe infection, which may have been associated with broader systemic inflammatory and metabolic responses in JBNU-22-N01-infected sows. Whether these systemic changes influence local immune competence at the endometrium remains an important question for future investigation.

This study has several limitations. First, the number of animals included at each necropsy time point was limited, which may reduce statistical power and constrain the generalizability of the findings. However, the major biological patterns were broadly consistent across multiple independent datasets, including maternal viral kinetics, tissue viral loads, fetal viral burden distribution, histopathology, immunohistochemistry, and gene-expression/cytokine analyses. Second, the transcriptional changes observed in the endometrium and fetal tissues should not be interpreted as direct proof that these molecular alterations alone caused specific morphological lesions leading to fetal death. Rather, these data are more appropriately viewed as indicators of altered barrier function, immune responses, and viral transmission dynamics at the maternal–fetal interface. In particular, the observed downregulation of junction-associated genes is interpreted as a plausible contributor to maternal–fetal interface dysfunction and increased susceptibility to transplacental viral passage, but not as definitive evidence of placental detachment or a direct structural cause of fetal demise.

The present findings also suggest several directions for future research. Higher-resolution approaches, including spatial transcriptomics, multiplex immunofluorescence, and in situ hybridization, will be valuable for defining the spatial relationships among viral localization, immune activation, and junction-associated gene dysregulation at the maternal–fetal interface. In addition, mechanistic studies using ex vivo explants or relevant cell-based models will be needed to determine whether the observed alterations in tight junction- and adherens junction-associated genes contribute to barrier dysfunction and increased susceptibility to transplacental viral transmission. Finally, studies incorporating larger animal cohorts and additional intermediate time points will help clarify the temporal sequence and relative contribution of maternal, placental, and fetal factors in the pathogenesis of fetal compromise and death.

**Figure 13:**
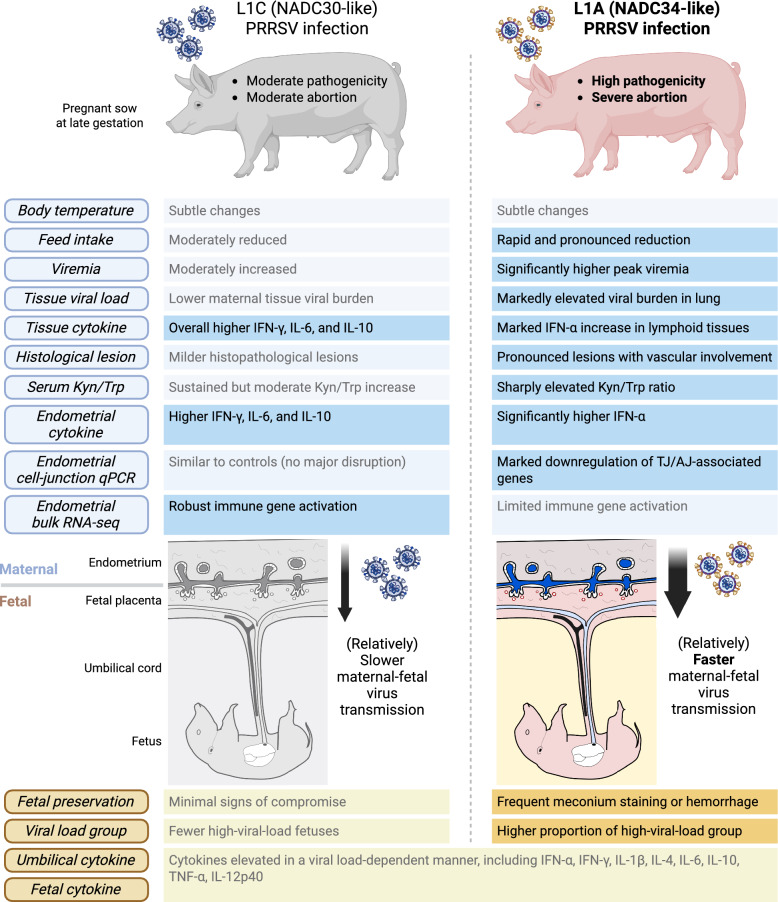
**Overview of strain-dependent pathological and immunological responses to PRRSV infection in pregnant sows. **A schematic comparison between NADC30-like (PJ73) and NADC34-like (JBNU-22-N01) PRRSV strains highlights key differences in maternal and fetal responses during late gestation infection. The NADC34-like strain induced higher maternal viremia, more severe histopathological lesions, and pronounced disruption of epithelial junctional genes in the endometrium, facilitating transplacental viral transmission. This resulted in greater fetal viral burden, frequent signs of compromise (e.g., meconium staining and hemorrhage), and cytokine upregulation in a viral load-dependent manner. By contrast, the NADC30-like strain elicited more controlled immune activation and preserved epithelial integrity, leading to milder fetal outcomes.

Despite the limited number of animals and the restricted scope of transcriptomic analysis to selected endometrial samples, this study provides new insights into the strain-specific immunopathogenic mechanisms of PRRSV at the maternal–fetal interface. Collectively, our findings support a model in which strain-dependent events at the maternal–fetal interface play a central role in determining fetal outcome during late-gestation PRRSV infection. In NADC34-like PRRSV infection, stronger systemic and local IFN-α responses, more pronounced maternal vascular pathology, higher endometrial viral burden, downregulation of tight and adherens junction-associated genes, and weaker local immune transcriptional activation were associated with more efficient maternal-to-fetal viral transmission, greater fetal viral burden, and more frequent fetal compromise. In contrast, NADC30-like PRRSV was associated with a comparatively more balanced local immune response and less severe disruption of maternal–fetal interface-associated transcriptional programs. Overall, these findings support a strain-dependent model of reproductive PRRS pathogenesis in which virological, immunological, and barrier-related processes interact to shape fetal outcome, rather than any single cytokine or molecular factor acting alone (Figure 13).

## Supplementary Information

**Additional file 1** **qPCR validation demonstrating identical amplification efficiencies between PRRSV strains.**

**Additional file 2** **Primers used in this study for qPCR.**

**Additional file 3** **PRRSV genomic RNA levels in pleural effusion of aborted fetuses at parturition. **Data represent individual fetuses per group.

**Additional file 4** **Tissue-specific comparison of cytokine levels in maternal tissues of PRRSV-infected sows.**

**Additional file 5** **Representative histopathological lesions in tissues of PRRSV-inoculated sows at 8 days post-challenge.**

**Additional file 6** **Strong positive correlations among viral loads in fetal compartments and associated tissues.**

**Additional file 7** **Quantitative regression analysis of PRRSV strain and viral load effects on endometrial gene expression.**

**Additional file 8.** **Quantitative regression dissection of PRRSV strain and viral load contributions to cytokine responses in maternal and fetal tissues.**

**Additional file 9** **GO term enrichment analysis.**

**Additional file 10** **KEGG pathway enrichment analysis.**

## Data Availability

The datasets used and/or analyzed during the current study are available from the corresponding author on reasonable request. The RNA-seq data have been deposited in NCBI BioProject under accession number PRJNA1370673(SRA accession number SRR36232060-SRR36232068)
