# Supplementary material for: Immunopathogenesis and pathological features of NADC34-like PRRSV infection in pregnant sows during late gestation
Source: Vet Res. 2026 Jul 24;57:138. doi: 10.1186/s13567-026-01792-0 (PMC13401299; doi:10.1186/s13567-026-01792-0)
Supplement: Supplementary file 1 — Additional file 1 qPCR validation demonstrating identical amplification efficiencies between PRRSV strains. [file 13567_2026_1792_MOESM1_ESM.pdf]

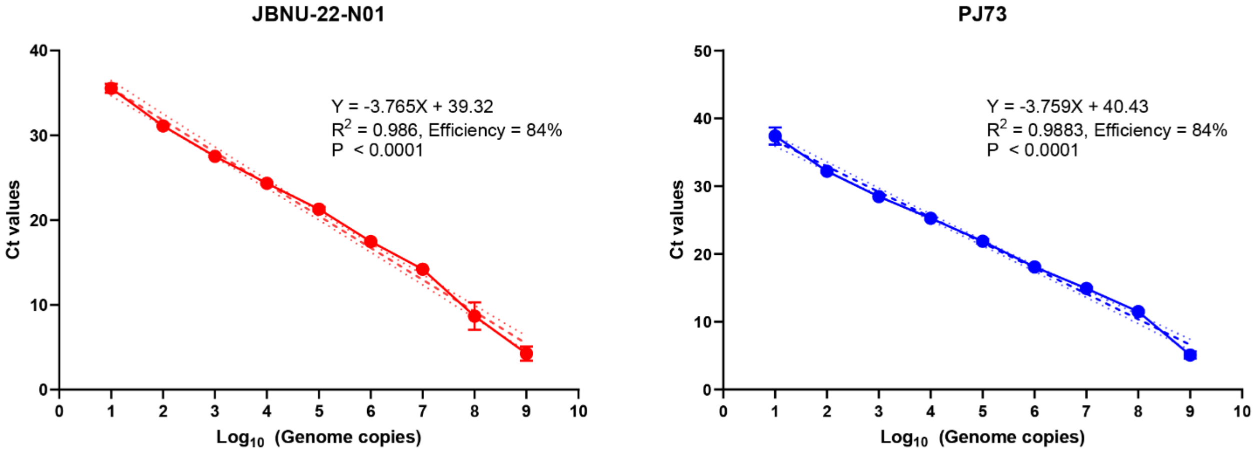

Supplementary Figure 1. qPCR validation demonstrating identical amplification efficiencies between PRRSV strains. Standard curves for (A) JBNU-22-N01 and (B) PJ73 represent linear relationships between log10 genome copies and Ct values. Serial 10-fold dilutions (10^1 to 10^9) were analyzed in triplicate. Both strains demonstrated identical amplification efficiencies (84%) with excellent linearity ($R^2 > 0.98$, $P < 0.0001$). Error bars represent standard error of the mean of replicate measurements.
