# Supplementary material for: Immunopathogenesis and pathological features of NADC34-like PRRSV infection in pregnant sows during late gestation
Source: Vet Res. 2026 Jul 24;57:138. doi: 10.1186/s13567-026-01792-0 (PMC13401299; doi:10.1186/s13567-026-01792-0)
Supplement: Supplementary file 2 — Additional file 2 Primers used in this study for qPCR. [file 13567_2026_1792_MOESM2_ESM.pdf]

Supplementary Table 1. Primers used in this study for qPCR

Target	Direction	Sequence (5'-3')	Amplicon (bp)	Remark	Sequence reference
HPRT (ref)	Forward	GGACTTGAATCATGTTTGTG	91	Housekeeping gene	Nygard et al., 2007 [50]
	Reverse	CAGATGTTTCCAAACTCAAC			
CLDN1	Forward	TGGAAGATGATGAGGTGCAG	95	Tight junction -related	Guidoni et al., 2022 [23]
	Reverse	CCATGCTGTGGCAACTAAGAT			
CLDN4	Forward	CAACTGCGTGGATGATGAGA	140	Tight junction -related	Pasternak et al., 2015 [47]
	Reverse	CCAGGGGATTGTAGAAGTCG			
CLDN5	Forward	CCTTCCTGGACCACAACATC	110	Tight junction -related	Pasternak et al., 2015 [47]
	Reverse	CACCGAGTCGTACACCTTGC			
CLDN6	Forward	CTTCATCGGCAACAGCATC	112	Tight junction -related	Pasternak et al., 2018 [48]
	Reverse	CAGCAGCGAGTCATACACCT			
CLDN10	Forward	TGGTTCCATATTTGCCCTGT	115	Tight junction -related	Guidoni et al., 2022 [23]
	Reverse	GCACAGCCCTGACAGTATGA			
TJP1	Forward	ACGGCGAAGGTAATTCAGTG	111	Tight junction -related	Pasternak et al., 2018 [48]
	Reverse	CTTCTCGGTTTGGTGGTCTG			
CDH1	Forward	TGAAGCCAAGCAGCAGTACA	86	Adherens junction -related	NM_001163060.1 (NCBI primer blast)
	Reverse	CAGTGGCCGTGGAAGTAGAG			
CXADR	Forward	GAAACCCTGGACTGGACGAG	121	Adherens junction -related	Kwon et al., 2016 [49]
	Reverse	GTAGCACCACTTGCAGAGGA			
PD1	Forward	AGCCCAAGCACTTCATCCTC	147	Immune checkpoints	Ruedas-Torres, I. et al. 2021 [28]
	Reverse	TGTGGAAGTCTCGTCCGTTG			
PDL1	Forward	GTGGAAAAATGTGGCAGCCG	140	Immune checkpoints	
	Reverse	TGCTTAGCCCTGACGAACTC			
ISG15	Forward	AGCAACGCCTATGAGGTCTG	107	Interferon-stimulated gene (ISG)	Kim et al., 2025 [65]
	Reverse	CCCTCGAAAGTCAGCCAGAA			
ISG12(A)	Forward	CCAAGATACTGGCGACAGGG	114	Interferon-stimulated gene (ISG)	
	Reverse	CGGTTAGGGCAGCCTTGAAT			
TREM2	Forward	CACACTCACCATTACGCTGC	125	Potential M2 macrophage marker	
	Reverse	GACCTTCTTGAGGGTGTCGG			
SPP1	Forward	TAATTCTGGCAGCTCGGAGG	108	Potential M2 macrophage marker	
	Reverse	TGTGGCGCTAGGAAAGTCTG			
C1R	Forward	CTCTCCCTGGACACAGAGTGG	89	Complement molecule	XM_021092408.1 (NCBI primer blast)
	Reverse	AGGAGGTATACGAGCCACATTTCT			
CD2	Forward	ATCCTGTGGAAGTGGAACACC	116	Pan-T cell marker	NM_213776.1 (NCBI primer blast)
	Reverse	ATATCCAGACCTTGCCCCGTA			
CXCL10	Forward	TGCAGCACCATGAACCAAAG	109	Chemokine	Kim et al., 2025 [65]
	Reverse	TGATGCAGGTACAGCGAACA			
MX1	Forward	AGTTACCGGGACAGCGAGAT	103	Interferon-stimulated gene	
	Reverse	GACTGATTCCCACGCCTTCC			
