# Supplementary material for: Immunopathogenesis and pathological features of NADC34-like PRRSV infection in pregnant sows during late gestation
Source: Vet Res. 2026 Jul 24;57:138. doi: 10.1186/s13567-026-01792-0 (PMC13401299; doi:10.1186/s13567-026-01792-0)
Supplement: Supplementary file 3 — Additional file 3 PRRSV genomic RNA levels in pleural effusion of aborted fetuses at parturition. Data represent individual fetuses per group. [file 13567_2026_1792_MOESM3_ESM.pdf]

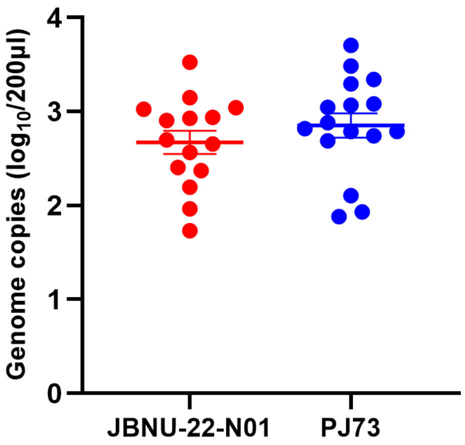

Supplementary Figure 2. PRRSV genomic RNA levels in pleural effusion of aborted fetuses at parturition. Data represent individual fetuses per group
