# Supplementary material for: Immunopathogenesis and pathological features of NADC34-like PRRSV infection in pregnant sows during late gestation
Source: Vet Res. 2026 Jul 24;57:138. doi: 10.1186/s13567-026-01792-0 (PMC13401299; doi:10.1186/s13567-026-01792-0)
Supplement: Supplementary file 4 — Additional file 4 Tissue-specific comparison of cytokine levels in maternal tissues of PRRSV-infected sows. [file 13567_2026_1792_MOESM4_ESM.pdf]

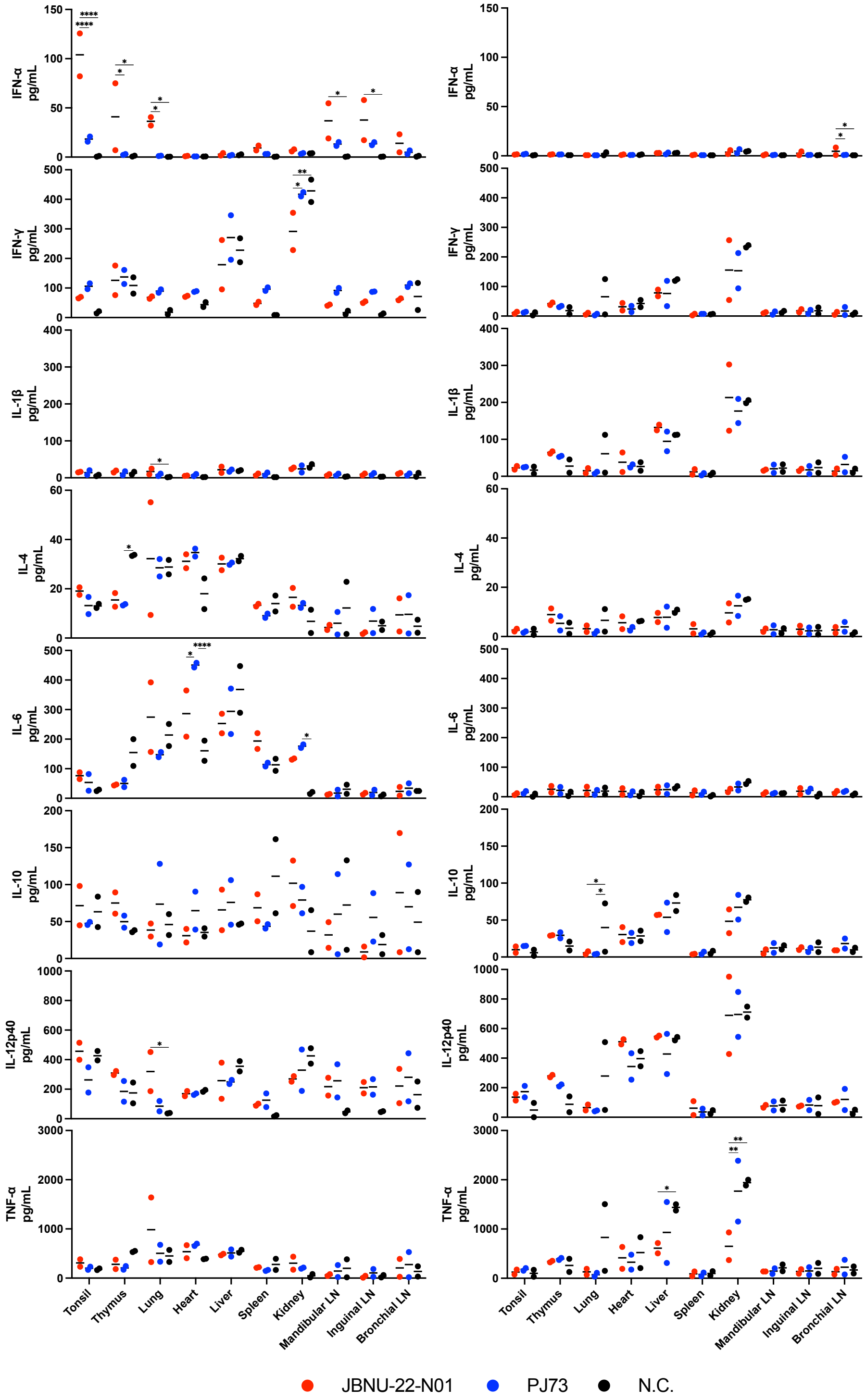

Supplementary Figure 3. Tissue-specific comparison of cytokine levels in maternal tissues of PRRSV-infected sows. Cytokine concentrations were measured in the indicated maternal tissues at each timepoint. Each dot represents an individual sample, and the horizontal line indicates the group mean. Statistical analysis was performed using two-way ANOVA with Tukey's multiple comparisons test. Statistical significance: $*p < 0.05$, $**p < 0.01$, $***p < 0.001$, $****p < 0.0001$.
