# Supplementary material for: Immunopathogenesis and pathological features of NADC34-like PRRSV infection in pregnant sows during late gestation
Source: Vet Res. 2026 Jul 24;57:138. doi: 10.1186/s13567-026-01792-0 (PMC13401299; doi:10.1186/s13567-026-01792-0)
Supplement: Supplementary file 5 — Additional file 5 Representative histopathological lesions in tissues of PRRSV-inoculated sows at 8 days post-challenge. [file 13567_2026_1792_MOESM5_ESM.pdf]

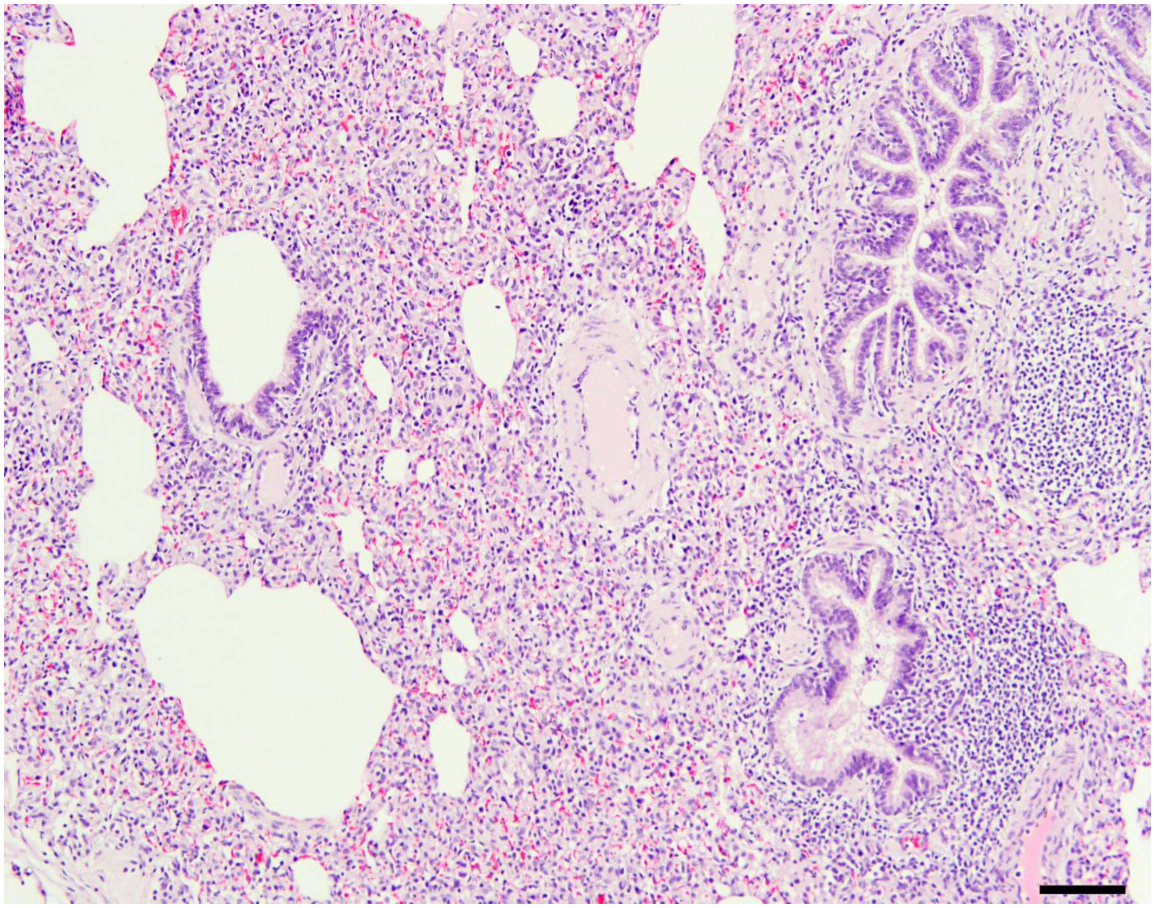

Supplementary Figure 4A. Interstitial pneumonia and perivascular cuffing in PJ73-infected lung tissue.

Interstitial pneumonia (score 2) at 8 days post-challenge (dpc), PJ73-infected sow. Note mild to moderate thickening of alveolar wall by the infiltration of mononuclear inflammatory cells with peribronchiolar and perivascular cuffing. H&E, Scale bar = 100 μm.

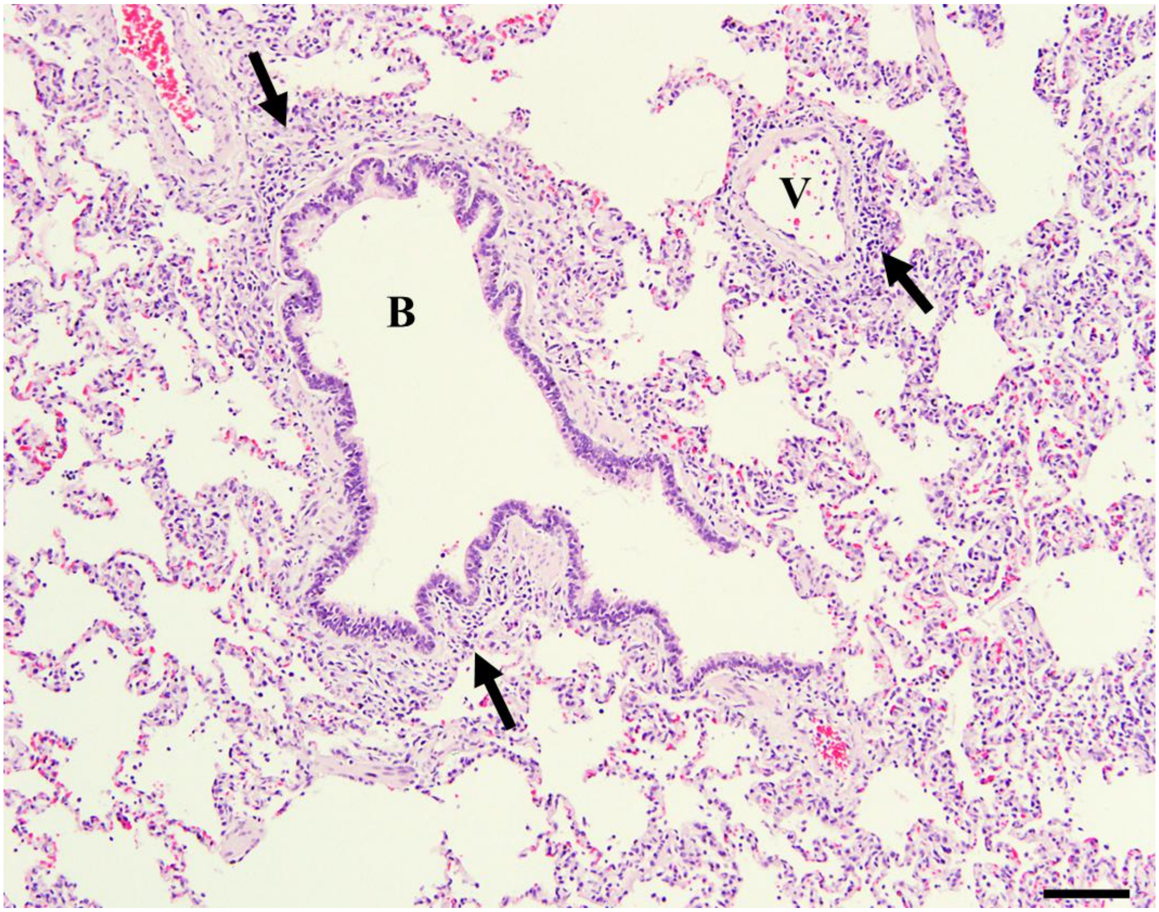

Supplementary Figure 4B. Peribronchiolar and perivascular cuffing in JBNU-22-N01-infected lung tissue.

Peribronchiolar and Perivascular cuffing (score 2) at 8 dpc, JBNU-22-N01-infected sow. Note the accumulation of mononuclear inflammatory cells (arrows) around the bronchiole (B) and pulmonary vessel (V). H&E, Scale bar = 100 ㎛.

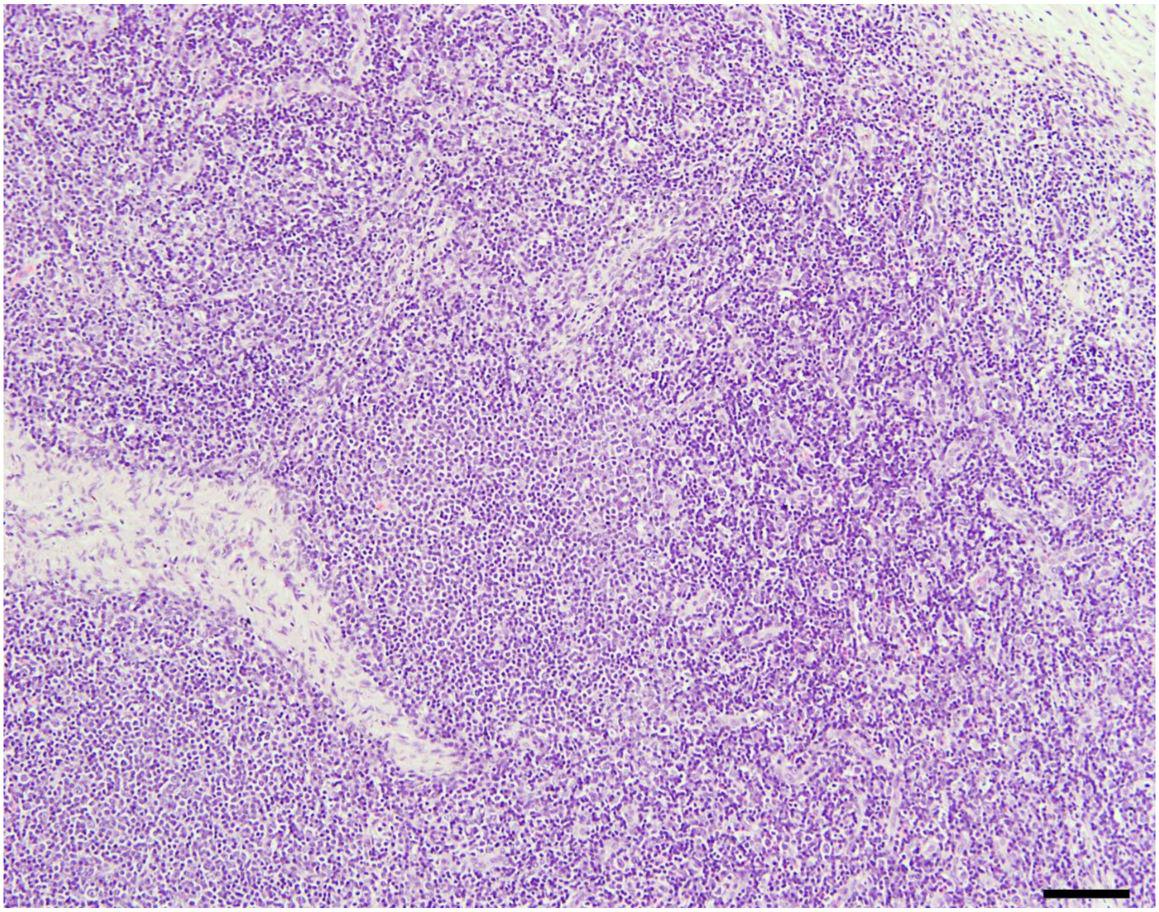

Supplementary Figure 4C. Paracortical hyperplasia in JBNU-22-N01-infected lymph node. Lymphoid hyperplasia (score 3) at 8dpc, JBNU-22-N01-infected sow. Note the expanded paracortical areas by increase number of lymphoid cells. H&E, Scale bar = 100 ㎛.

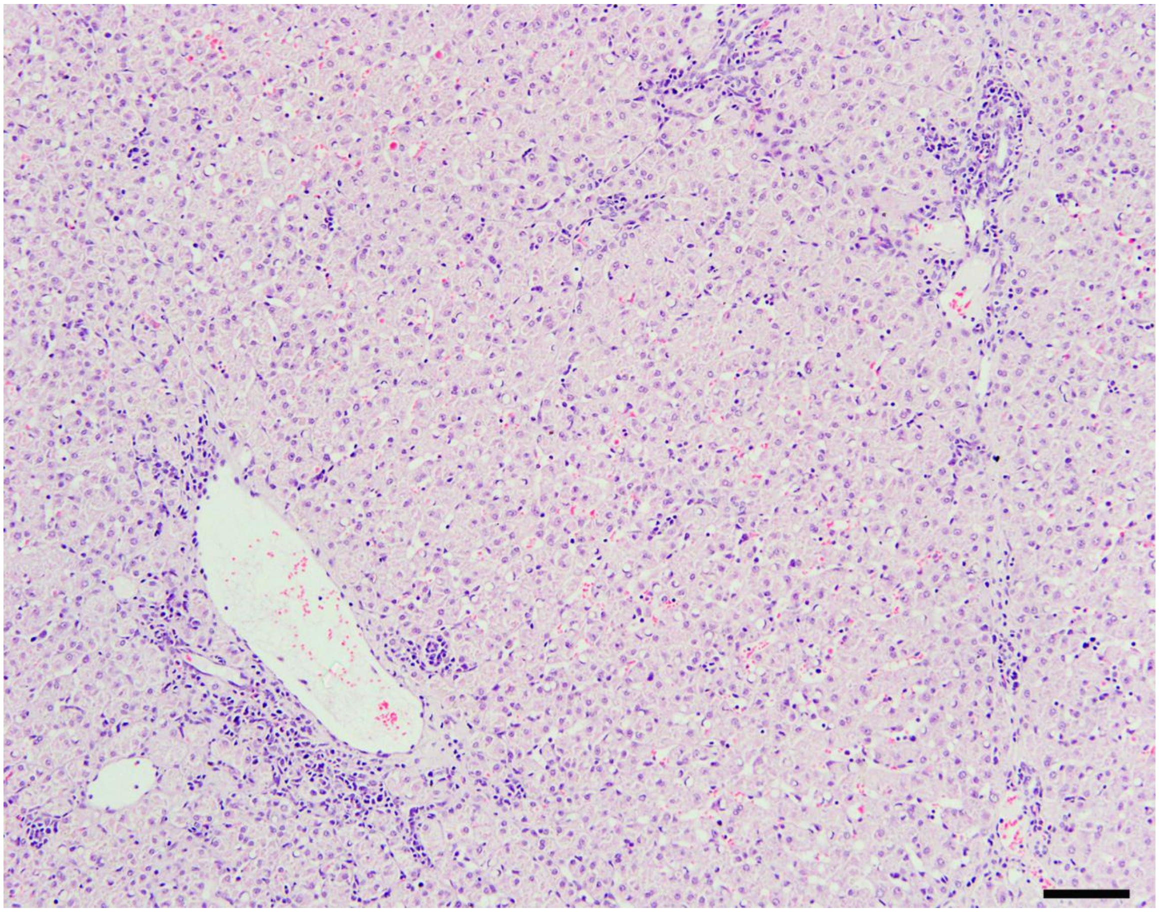

Supplementary Figure 4D. Portal inflammation in JBNU-22-N01-infected liver tissue. Multifocal inflammatory reaction including portal areas (score 2) at 8dpc, JBNU-22-N01-infected sow. Most of the infiltrated cells were identified as a mononuclear cell such as lymphocytes and plasma cells. H&E, Scale bar = 100 ㎛.

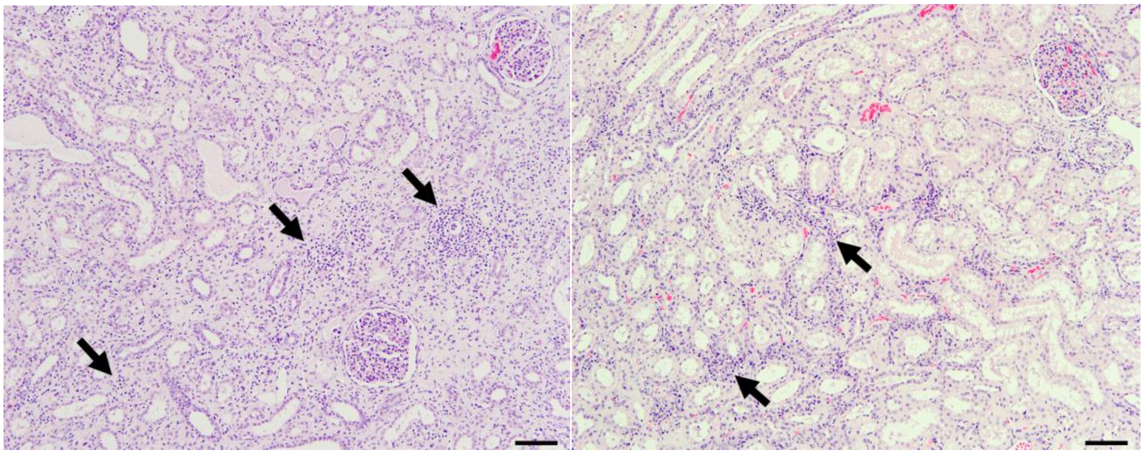

Supplementary Figure 4E. Interstitial nephritis in PRRSV-infected kidney tissue.
(Left) Interstitial nephritis (score 2) at 8dpc, JBNU-22-N01-infected sow. Note the multifocal infiltration of mononuclear inflammatory cells in the renal cortex (arrow). (Right) Interstitial nephritis (score 2), PJ73-infected sow. Note the multifocal infiltration of mononuclear inflammatory cells in the renal cortex (arrows). H&E, Scale bar = 100 ㎛.

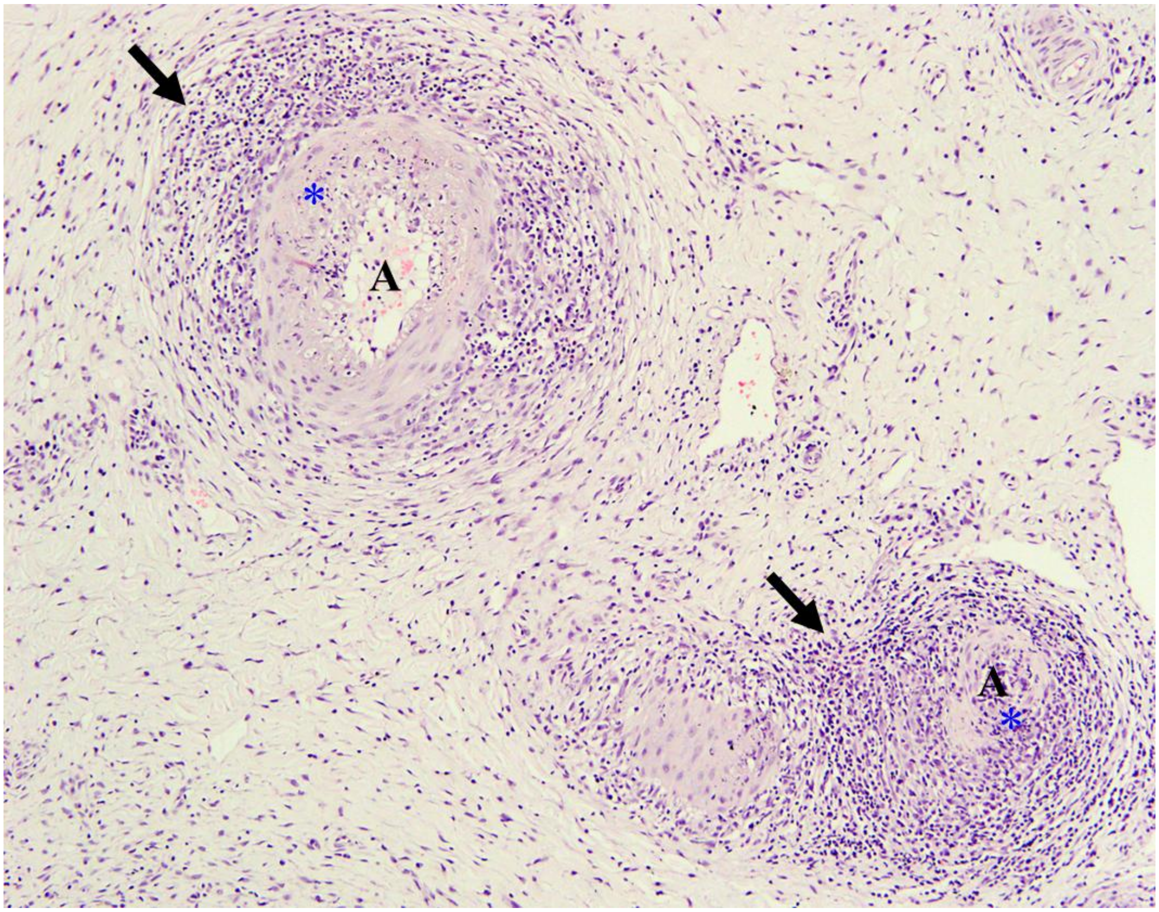

Supplementary Figure 4F. Severe arteritis in JBNU-22-N01-infected kidney tissue.
Severe arteritis/periarteritis with fibrinoid degeneration (score 4) at 8dpc, JBNU-22-N01-infected sow. Note the accumulation of numerous mononuclear inflammatory cells around the arteries (A, arrows) and within the media (asterisks). H&E, Scale bar = 100 ㎛.

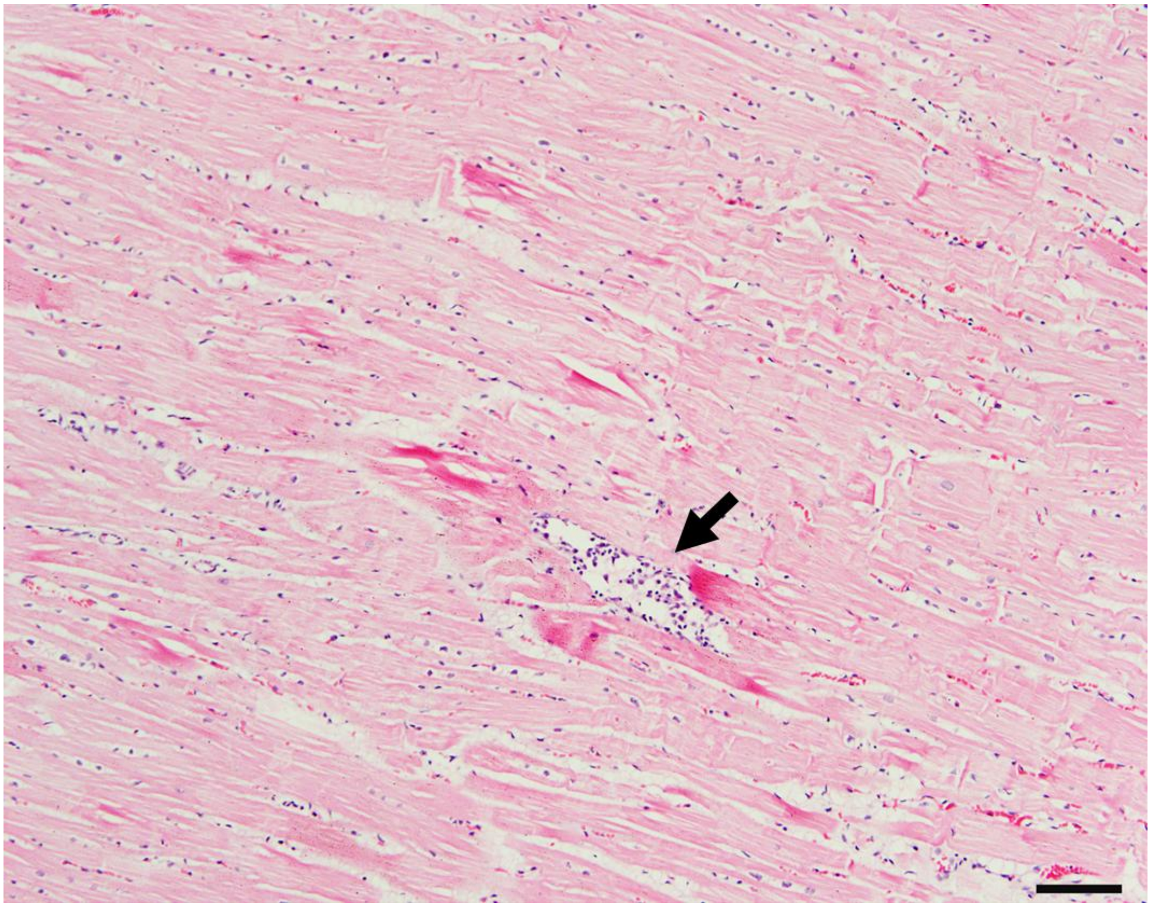

Supplementary Figure 4G. Myocarditis in JBNU-22-N01-infected heart tissue.

Myocarditis (score 1) at 8dpc, JBNU-22-N01-infected sow. Note the focal infiltration of mononuclear inflammatory cells in the myocardium (arrow). H&E, Scale bar = 100 μm.
