# Supplementary material for: Immunopathogenesis and pathological features of NADC34-like PRRSV infection in pregnant sows during late gestation
Source: Vet Res. 2026 Jul 24;57:138. doi: 10.1186/s13567-026-01792-0 (PMC13401299; doi:10.1186/s13567-026-01792-0)
Supplement: Supplementary file 6 — Additional file 6 Strong positive correlations among viral loads in fetal compartments and associated tissues. [file 13567_2026_1792_MOESM6_ESM.pdf]

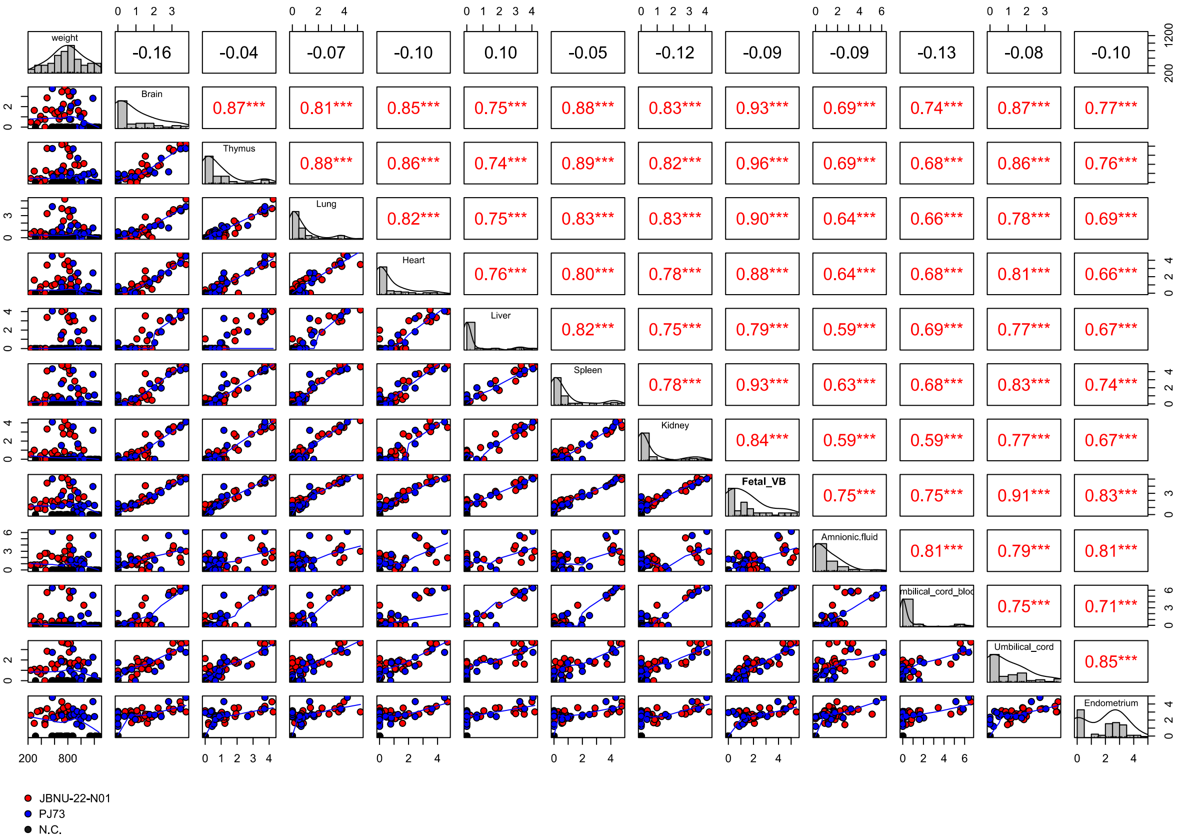

Supplementary Figure 5. Strong positive correlations among viral loads in fetal compartments and associated tissues. Spearman correlation matrix of viral loads (log10 genome copies / 200μL) measured in individual fetal tissues, including the brain, thymus, lung, heart, liver, spleen, kidney, as well as amniotic fluid, umbilical cord blood, umbilical cord, and the associated endometrium. Viral loads across these compartments demonstrated strong positive correlations, supporting the integration of individual tissue viral loads into a single composite metric, referred to as Fetal Viral Burden (Fetal VB). This aggregated measure was used to represent the overall viral burden for each fetus in subsequent analyses.
