# Supplementary material for: Immunopathogenesis and pathological features of NADC34-like PRRSV infection in pregnant sows during late gestation
Source: Vet Res. 2026 Jul 24;57:138. doi: 10.1186/s13567-026-01792-0 (PMC13401299; doi:10.1186/s13567-026-01792-0)
Supplement: Supplementary file 7 — Additional file 7 Quantitative regression analysis of PRRSV strain and viral load effects on endometrial gene expression. [file 13567_2026_1792_MOESM7_ESM.pdf]

A

$$Cytokine\ (pg/mL) \sim \beta_0 + \beta_1 \times strain + \beta_2 \times viral\ load$$

Infection group (strain): ● JBNU-22-N01 ● PJ73 ● N.C.

MFI-Fetus Viral Load: ● High ● High-middle ● Middle ● Middle-low ● Low ● Non-infected

B Endometrium

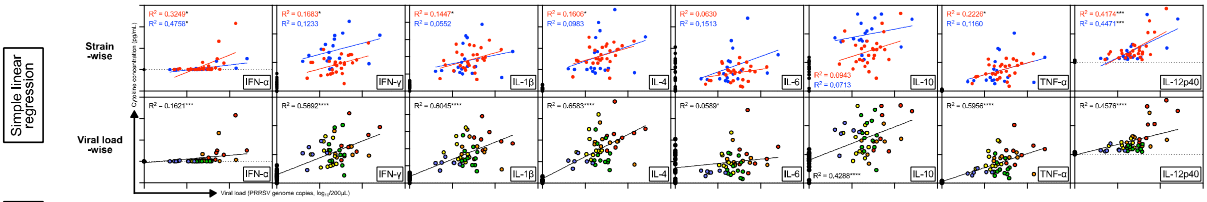

Multiple linear regression	IFN-α	IFN-γ	IL-1β	IL-4	IL-6	IL-10	TNF-α	IL-12p40
**Strain effect (ANOVA)**	$p = 0.0015^{**}$	$p < 0.0001^{****}$	$p = 0.1832$	$p = 0.1390$	$p = 0.0822$	$p < 0.0001^{****}$	$p = 0.7926$	$p = 0.0008^{***}$
N.C. vs JBNU-22-N01	$p = 0.0027^{**}$	$p = 0.4876$	$p = 0.0734$	$p = 0.0554$	$p = 0.0906$	$p = 0.6758$	$p = 0.5098$	$p = 0.0003^{**}$
N.C. vs PJ73	$p = 0.0004^{***}$	$p = 0.0014^{**}$	$p = 0.0758$	$p = 0.0536$	$p = 0.4140$	$p = 0.0095^{**}$	$p = 0.5094$	$p = 0.0002^{***}$
**Viral load effect (ANOVA)**	$p < 0.0001^{****}$	$p = 0.0023^{**}$	$p = 0.0139^{*}$	$p = 0.0046^{**}$	$p = 0.0337^{*}$	$p = 0.0300^{*}$	$p = 0.0010^{**}$	$p < 0.0001^{****}$
**Goodness of Fit**	$R^2 = 0.3117$	$R^2 = 0.7167$	$R^2 = 0.6243$	$R^2 = 0.6781$	$R^2 = 0.1275$	$R^2 = 0.5838$	$R^2 = 0.5984$	$R^2 = 0.5637$

C Umbilical cord

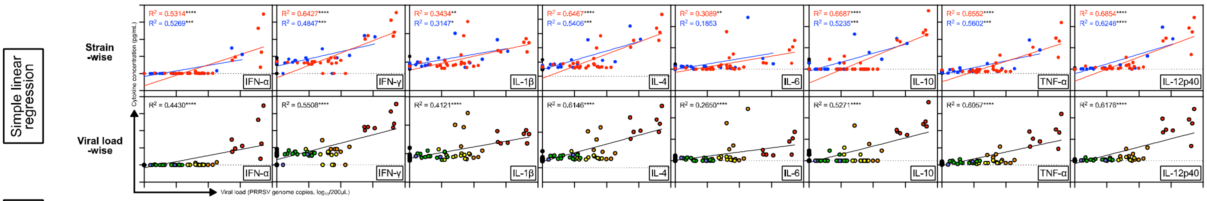

Multiple linear regression	IFN-α	IFN-γ	IL-1β	IL-4	IL-6	IL-10	TNF-α	IL-12p40
**Strain effect (ANOVA)**	$p = 0.0045^{**}$	$p = 0.0277^{*}$	$p = 0.4421$	$p = 0.0112^{*}$	$p = 0.2823$	$p = 0.0003^{***}$	$p = 0.0052^{**}$	$p = 0.0003^{***}$
N.C. vs JBNU-22-N01	$p = 0.0012^{**}$	$p = 0.0096^{**}$	$p = 0.3387$	$p = 0.0037^{**}$	$p = 0.1261$	$p < 0.0001^{****}$	$p = 0.0013^{**}$	$p < 0.0001^{****}$
N.C. vs PJ73	$p = 0.0137^{*}$	$p = 0.2630$	$p = 0.9463$	$p = 0.2045$	$p = 0.5297$	$p = 0.0234^{*}$	$p = 0.0280^{*}$	$p = 0.0083^{**}$
**Viral load effect (ANOVA)**	$p < 0.0001^{****}$	$p < 0.0001^{****}$	$p < 0.0001^{****}$	$p < 0.0001^{****}$	$p < 0.0001^{****}$	$p < 0.0001^{****}$	$p < 0.0001^{****}$	$p < 0.0001^{****}$
**Goodness of Fit**	$R^2 = 0.5272$	$R^2 = 0.5971$	$R^2 = 0.4264$	$R^2 = 0.6636$	$R^2 = 0.2927$	$R^2 = 0.6297$	$R^2 = 0.6637$	$R^2 = 0.7001$

D Fetal lung

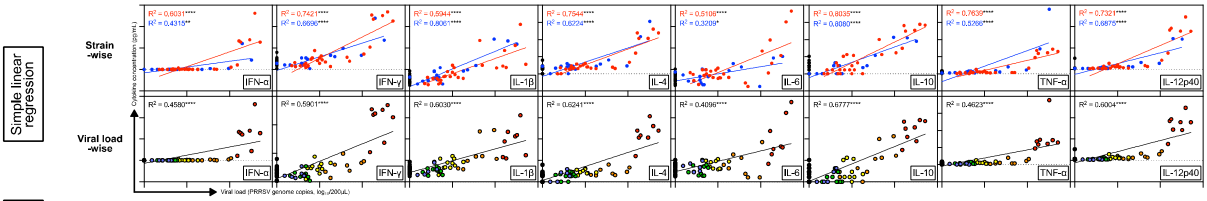

Multiple linear regression	IFN-α	IFN-γ	IL-1β	IL-4	IL-6	IL-10	TNF-α	IL-12p40
**Strain effect (ANOVA)**	$p = 0.0021^{**}$	$p < 0.0001^{****}$	$p = 0.0002^{***}$	$p = 0.0001^{***}$	$p = 0.0391^{*}$	$p < 0.0001^{****}$	$p < 0.0001^{****}$	$p < 0.0001^{****}$
N.C. vs JBNU-22-N01	$p = 0.0031^{**}$	$p < 0.0001^{****}$	$p < 0.0001^{****}$	$p < 0.0001^{****}$	$p = 0.0520$	$p < 0.0001^{****}$	$p < 0.0001^{****}$	$p < 0.0001^{****}$
N.C. vs PJ73	$p = 0.0007^{***}$	$p < 0.0001^{****}$	$p = 0.0118^{*}$	$p = 0.0009^{***}$	$p = 0.0120^{*}$	$p < 0.0001^{****}$	$p = 0.0068^{**}$	$p < 0.0001^{****}$
**Viral load effect (ANOVA)**	$p < 0.0001^{****}$	$p < 0.0001^{****}$	$p < 0.0001^{****}$	$p < 0.0001^{****}$	$p < 0.0001^{****}$	$p < 0.0001^{****}$	$p < 0.0001^{****}$	$p < 0.0001^{****}$
**Goodness of Fit**	$R^2 = 0.5504$	$R^2 = 0.7046$	$R^2 = 0.6917$	$R^2 = 0.7139$	$R^2 = 0.4648$	$R^2 = 0.7758$	$R^2 = 0.5955$	$R^2 = 0.7105$

E

	IFN-α	IFN-γ	IL-1β	IL-4	IL-6	IL-10	TNF-α	IL-12p40
**Endometrium**	● (cyan) ● (magenta)	● (cyan) ● (magenta)		● (magenta)		● (cyan)	● (magenta)	● (cyan) ● (magenta)
**Umbilical cord**	● (cyan) ● (magenta)	● (magenta)	● (magenta)	● (magenta)	● (magenta)	● (cyan) ● (magenta)	● (cyan) ● (magenta)	● (cyan) ● (magenta)
**Fetal lung**	● (cyan) ● (magenta)	● (cyan) ● (magenta)	● (cyan) ● (magenta)	● (cyan) ● (magenta)	● (magenta)	● (cyan) ● (magenta)	● (cyan) ● (magenta)	● (cyan) ● (magenta)

● (cyan) PRRSV strain-dependent
● (magenta) Absolute viral load-dependent

Supplementary Figure 6. Quantitative regression analysis of PRRSV strain and viral load effects on endometrial gene expression. (A) Multiple linear regression (MLR) model used to assess the independent effects of PRRSV strain and viral load on gene expression levels, formulated as: Gene expression (log2 fold change, -ΔΔCt) = $\beta_0 + \beta_1 \times \text{strain} + \beta_2 \times \text{viral load}$. (B–C) Summary of regression analyses for gene expression levels measured in the endometrium. (B) Cell junction-related genes, including tight junction-associated genes (CLDN1, CLDN4, CLDN5, CLDN6, CLDN10, TJP1) and adherens junction components (CDH1, CXADR). (C) Immune-related genes, including immune checkpoint molecules (PD1, PDL1), interferon-stimulated genes (ISG15, ISG12[A]), and macrophage markers (TREM2, SPP1). For each gene, simple linear regression analyses were performed to examine the correlation between gene expression levels and viral loads, stratified by strain group (strain-wise) and viral load category (viral load-wise), with R-squared values indicating the strength of association. In addition, MLR outcomes for each gene are presented, including the statistical significance of strain and viral load effects and model goodness-of-fit. (D) Summary of classification of gene expression patterns as strain-dependent or absolute viral load-dependent, based on the MLR analyses. Genes were designated as strain-dependent when the strain effect was significant at $p < 0.01$, and as absolute viral load-dependent when the viral load effect was significant at $p < 0.01$.
