# Supplementary material for: Immunopathogenesis and pathological features of NADC34-like PRRSV infection in pregnant sows during late gestation
Source: Vet Res. 2026 Jul 24;57:138. doi: 10.1186/s13567-026-01792-0 (PMC13401299; doi:10.1186/s13567-026-01792-0)
Supplement: Supplementary file 8 — Additional file 8. Quantitative regression dissection of PRRSV strain and viral load contributions to cytokine responses in maternal and fetal tissues. [file 13567_2026_1792_MOESM8_ESM.pdf]

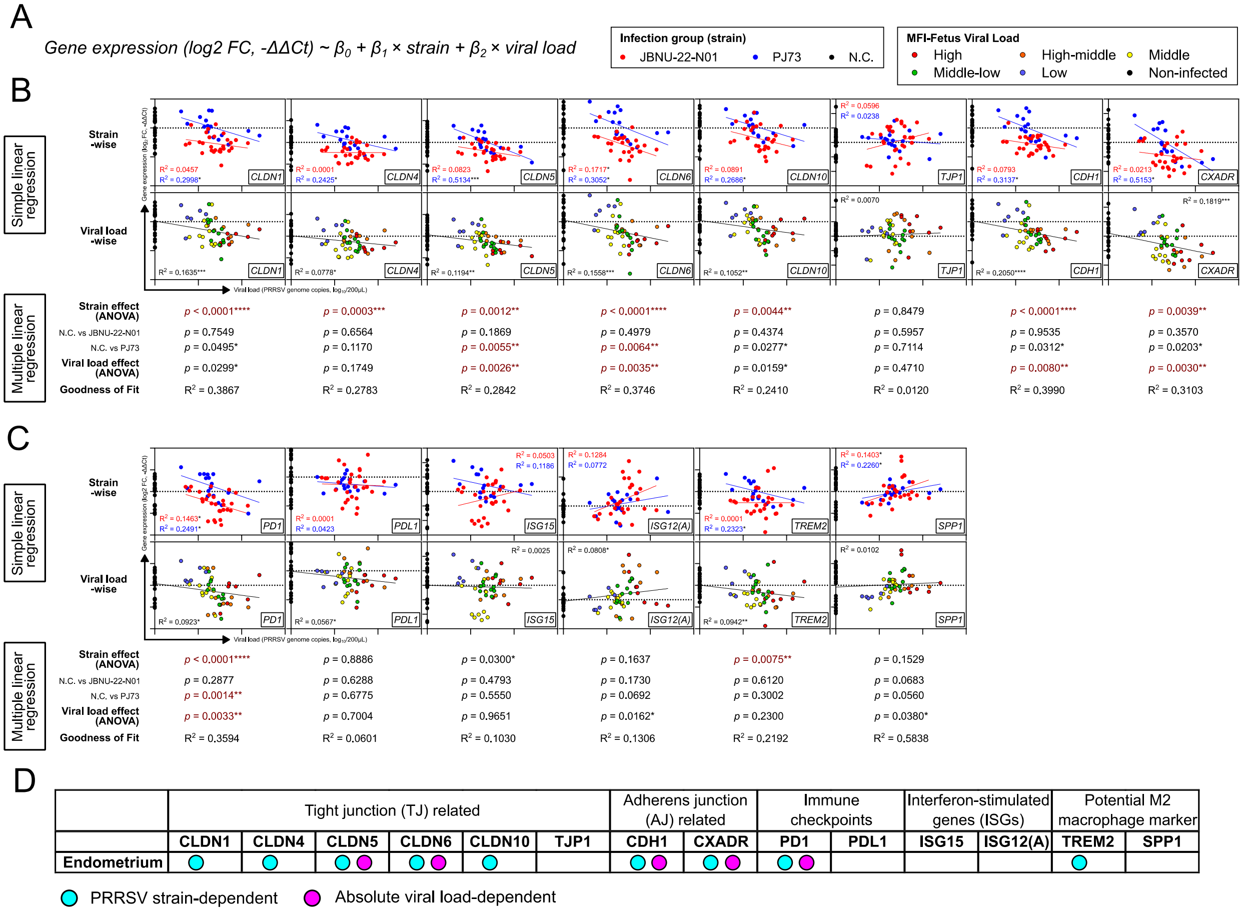

Supplementary Figure 7. Quantitative regression dissection of PRRSV strain and viral load contributions to cytokine responses in maternal and fetal tissues. (A) Multiple linear regression (MLR) model used to assess the independent effects of PRRSV strain and viral load on cytokine concentrations, formulated as: Cytokine concentration (pg/mL) = $\beta_0 + \beta_1 \times$ strain + $\beta_2 \times$ viral load. (B-D) Summary of the regression analyses for cytokines measured in the (B) endometrium, (C) umbilical cord, and (D) fetal lungs, respectively. For each tissue, simple linear regression analyses were performed to examine the correlation between cytokine concentrations and viral loads, stratified by strain group (strain-wise) and viral load category (viral load-wise), with R-squared values indicating the strength of association. In addition, MLR outcomes for each cytokine are presented, including the statistical significance of strain and viral load effects and model goodness-of-fit. (E) Summary of classification of cytokine responses as strain-dependent or absolute viral load-dependent, based on the MLR analyses. Cytokines were designated as strain-dependent when the strain effect was significant at $p < 0.01$, and as absolute viral load-dependent when the viral load effect was significant at $p < 0.01$.
