# Supplementary material for: Immunopathogenesis and pathological features of NADC34-like PRRSV infection in pregnant sows during late gestation
Source: Vet Res. 2026 Jul 24;57:138. doi: 10.1186/s13567-026-01792-0 (PMC13401299; doi:10.1186/s13567-026-01792-0)
Supplement: Supplementary file 9 — Additional file 9 GO term enrichment analysis. [file 13567_2026_1792_MOESM9_ESM.pdf]

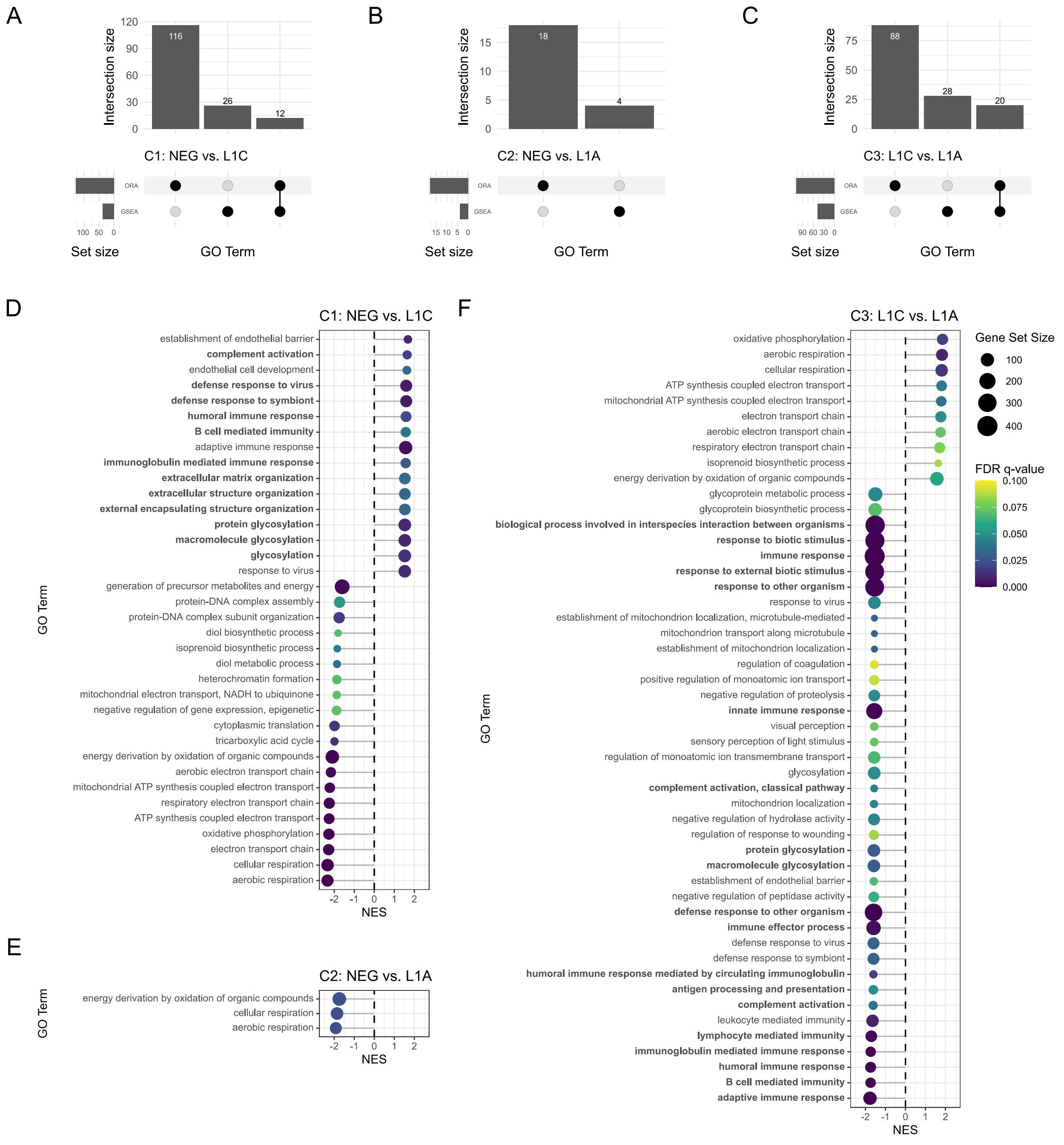

Supplementary Figure 8. GO term enrichment analysis. (A–C) UpSet plots showing overlap between Gene Ontology (GO) terms enriched by over-representation analysis (ORA) and gene set enrichment analysis (GSEA) for each comparison. (D–F) Dot plots of GSEA-enriched biological process (BP) GO terms: (D) NEG vs. L1C_MVL (C1), (E) NEG vs. L1A_MVL (C2), and (F) L1C_MVL vs. L1A_MVL (C3).
