# Supplementary material for: Immunopathogenesis and pathological features of NADC34-like PRRSV infection in pregnant sows during late gestation
Source: Vet Res. 2026 Jul 24;57:138. doi: 10.1186/s13567-026-01792-0 (PMC13401299; doi:10.1186/s13567-026-01792-0)
Supplement: Supplementary file 10 — Additional file 10 KEGG pathway enrichment analysis. [file 13567_2026_1792_MOESM10_ESM.pdf]

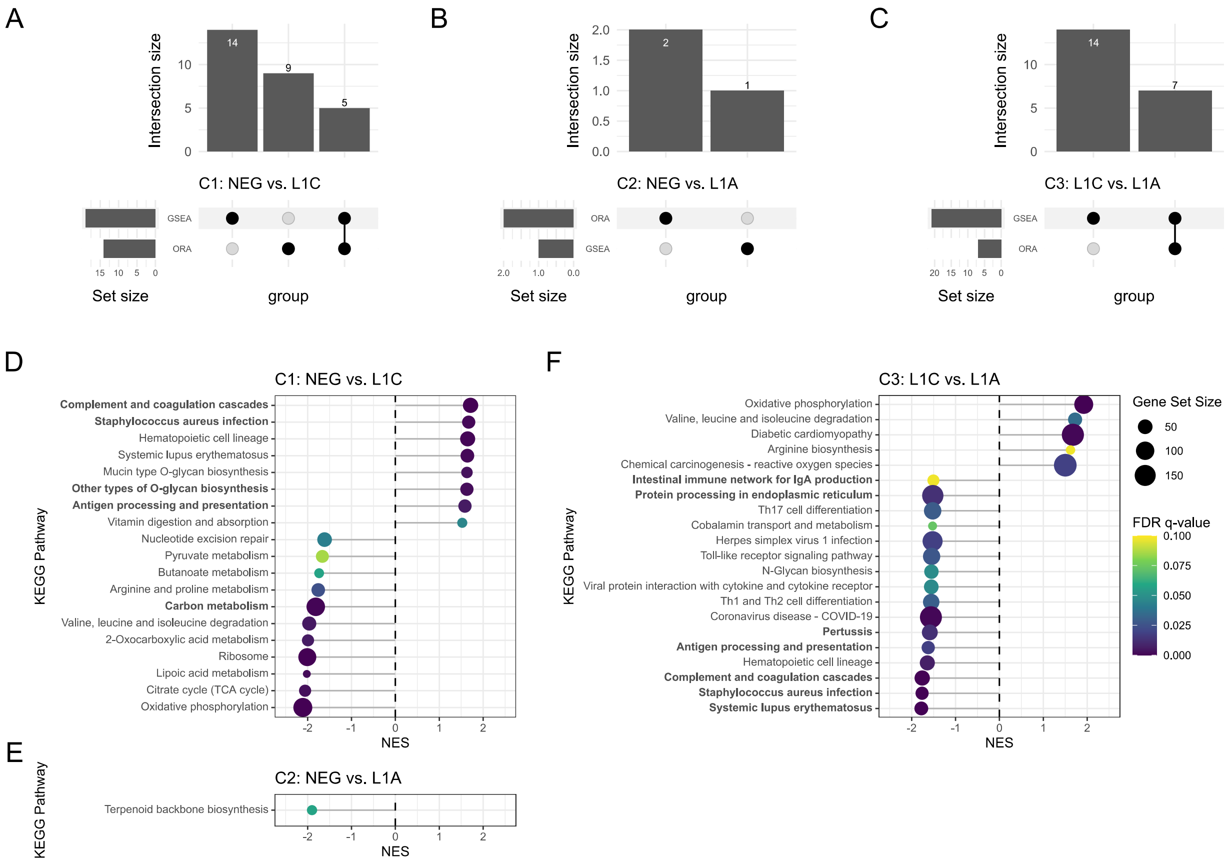

Supplementary Figure 9. KEGG pathway enrichment analysis. (A–C) UpSet plots showing overlap between ORA and GSEA results for KEGG pathways across three comparisons. (D–F) Dot plots representing GSEA-enriched KEGG pathways: (D) NEG vs. L1C_MVL (C1), (E) NEG vs. L1A_MVL (C2), and (F) L1C_MVL vs. L1A_MVL (C3). Immune-related pathways are strongly enriched in L1C_MVL samples.
